# Divergent cytokine and transcriptional signatures control functional T follicular helper cell heterogeneity

**DOI:** 10.1038/s41590-025-02258-9

**Published:** 2025-09-09

**Authors:** Lennard Dalit, Chin Wee Tan, Amania A. Sheikh, Ryan Munnings, Lauren J. Howson, Carolina Alvarado, Tabinda Hussain, Aidil Zaini, Lucy Cooper, Alana Kirn, Lauren Hailes, Angela Nguyen, Bailey E. Williams, Ming Z. M. Zheng, Carolien E. van de Sandt, Laura K. Mackay, Katie L. Flanagan, Katherine Kedzierska, Nicola Harris, Jennifer A. Juno, Colby Zaph, Nicole L. La Gruta, Melissa J. Davis, Stephen L. Nutt, Kim L. Good-Jacobson, Vanessa L. Bryant, Joanna R. Groom

**Affiliations:** 1 Walter and Eliza Hall Institute of Medical Research, Parkville, Victoria Australia; 2 Department of Medical Biology, University of Melbourne, Parkville, Victoria Australia; 3 Frazer Institute, Faculty of Medicine, The University of Queensland, Brisbane, Queensland Australia; 4 Department of Clinical Immunology & Allergy, Royal Melbourne Hospital, Parkville, Victoria Australia; 5 Department of Biochemistry and Molecular Biology, Immunity Program, Biomedicine Discovery Institute Monash University, Clayton, Victoria Australia; 6 Department of Immunology and Pathology, Monash University, Melbourne, Victoria Australia; 7 Department of Microbiology and Immunology, The University of Melbourne at the Peter Doherty Institute for Infection and Immunity, Melbourne, Victoria Australia; 8 School of Health Sciences and School of Medicine, University of Tasmania, Launceston, Tasmania Australia; 9 School of Health and Biomedical Science, RMIT University, Melbourne, Victoria Australia; 10 Centre for Infectious Diseases and Microbiology, Westmead Hospital, Sydney, New South Wales Australia; 11 School of Biomedicine, Faculty of Health and Medical Sciences, The University of Adelaide, Adelaide, South Australia Australia

**Keywords:** Lymphocyte differentiation, Cytokines, Gene regulation in immune cells

## Abstract

CD4^+^ T follicular helper (T_FH_) cells support tailored B cell responses against multiple classes of pathogens. To reveal how diverse T_FH_ phenotypes are established, we profiled mouse T_FH_ cells in response to viral, helminth and bacterial infection. We identified a core T_FH_ signature that is distinct from CD4^+^ T follicular regulatory and effector cells and identified pathogen-specific transcriptional modules that shape T_FH_ function. Cytokine-transcriptional T_FH_ programming demonstrated that type I interferon and TGFβ signaling direct individual T_FH_ phenotypes to instruct B cell output. Cytokine-directed T_FH_ transcriptional phenotypes are shared within human germinal centers, but distinct T_FH_ phenotypes dominate between donors and following immune challenge or in antibody-mediated disease. Finally, we identified new cell surface markers that align with distinct T_FH_ phenotypes. Thus, we provide a comprehensive resource of T_FH_ diversity in humans and mice to enable immune monitoring during infection and disease and to inform the development of context-specific vaccines.

## Abstract (web summary)

Dalit, Tan and colleagues provide a multiomic profile of T follicular helper (T_FH_) cells responses to diverse pathogens, revealing a blueprint for transcriptional flexibility and new tools to interrogate T_FH_ heterogeneity in mice and humans.

## Main

Flexibility of the immune system ensures the clearance of, and generation of memory against a wide range of pathogen classes, such as viral, bacterial, fungal and helminth infections^1,2^. Environmental cues, such as infection route, expression of pathogen-associated molecular pattern molecules and antigen affinity and availability, are integrated to impact the generation of specialized CD4^+^ T helper (T_H_) cells that enable pathogen-specific adaptive immunity^3–7^. Accordingly, T_H_1 cells produce interferon (IFN)γ and tumor necrosis factor (TNF) to assist in antiviral responses, T_H_2 cells mediate protection against helminth infection via the production of interleukin (IL)-4, IL-5 and IL-13, and T_H_17 cells secrete IL-17 family cytokines to protect against bacteria and fungi^1,8^. In addition to the differentiation of these CD4^+^ T_H_ effector (T_eff_) cell subsets, CD4^+^ T_FH_ cells are generated to instruct humoral immunity by promoting the germinal center (GC) reaction and differentiation of T-dependent memory B cells and plasma cells, a process dependent on the cardinal T_FH_ cell cytokines IL-21 and IL-4 (refs. ^9–11^). In contrast to T_eff_ cells, which, despite their plasticity, are honed to defend against particular classes of pathogens, T_FH_ cells drive B cell responses across multiple settings while retaining the ability for context-specific tailoring^1,8,9,12^. Thus, T_FH_ cells are induced by all categories of pathogens and licensed vaccines, and influence the pathogenesis of diseases such as immunodeficiency, autoimmunity, asthma, allergies and cancer^9,12^. How this exceptional array of T_FH_ functions is orchestrated is not clear.

Much of the knowledge on T_FH_ differentiation has focused on the bifurcation model, where T_FH_ differentiation contrasts with that of T_H_1, T_H_2 or T_H_17 cells^9,13,14^; however, this model has been questioned and additional complexity has been proposed to incorporate T_FH_ functional diversity^12,15,16^. While multiple cytokine signaling pathways regulate T_FH_ fate, the role these mediators play in tailoring pathogen-specific T_FH_ phenotypes beyond this branch point is lacking^17–24^.

Evidence of T_FH_ heterogeneity has been assessed by the distinct expression of transcriptional regulators and varied production of T_H_ cytokines and chemokine receptor surface expression^12,15^. The T_FH_ lineage-defining transcription factor B cell lymphoma (Bcl)-6 can be coexpressed with transcription factors that define T_H_1, T_H_2 and T_H_17 differentiation, namely, T-bet, GATA3 and RORγt^9,12,15,17,25^. Aligned with these borrowed transcription factors, T_FH_ cells can produce the cytokines IFN-γ, IL-4, IL-10 and IL-17 to tailor B cell responses and dictate the class-switch isoform of high-affinity antibodies^11,26^. In humans, circulating memory T_FH_ (cT_FH_) cells are identified via the expression of chemokine receptors, where CXCR3^+^ cT_FH_ cells resemble T_H_1 cells, CXCR3^−^CCR6^−^ cells resemble T_H_2 cells and CCR6^+^ cells resemble T_H_17 cells^12,27–29^. Immune profiling of specific cT_FH_ populations is correlated with disease and protective vaccine outcomes^9,30–32^. Although T_FH_ cells can parallel T_eff_ cells at multiple levels, the extent of T_FH_ heterogeneity or how distinct T_FH_ phenotypes are established remains unclear.

In this study, we transcriptionally profiled a spectrum of T_FH_ phenotypic states induced by multiple pathogens, assessing viral, helminth and bacterial infections. By combining T_FH_ cell profiles across pathogens, we established a core T_FH_ signature that was separate from that of T follicular regulatory (T_FR_) cells and T_eff_ cells. In addition to T_FH_ identity, distinct T_FH_ phenotypes were directed by cytokine pathways to enable tailoring of B cell responses. These cytokine-directed transcriptional modules were evident in human tonsils and discriminated T_FH_ phenotypes present in clinical datasets. In combination, we present a resource to understand T_FH_ phenotype heterogeneity and plasticity; monitor T_FH_ dynamics during infection, vaccination, antibody deficiency and antibody-mediated disease; and reveal opportunities to fine-tune humoral responses for vaccination.

## Results

### Diverse pathogen challenges induce heterogeneous T_FH_ cells

**Fig. 1:**
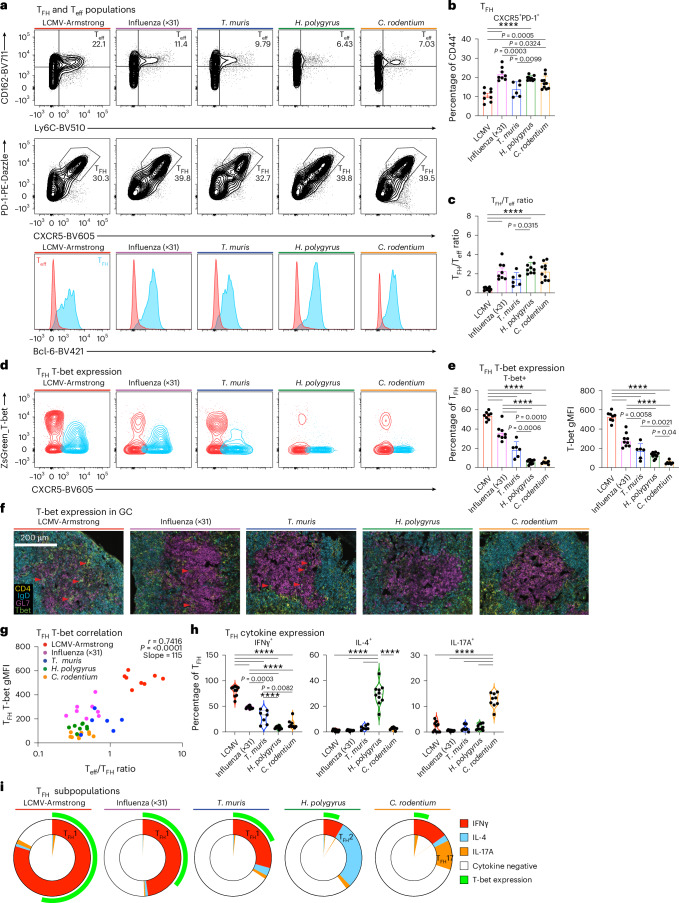
Functionally heterogeneous T_FH_ phenotypes are induced by diverse pathogen infections. **a**–**i**, Analysis of draining lymph node cells from wild-type (**a**–**c**,**h**,**i**) and ZsGreen_T-bet reporter (**d**–**g**) mice infected with the indicated pathogens at the early peak GC response (LCMV day 12, influenza day 10, *T.* *muris* day 21, *H.* *polygyrus* day 12 and *C.* *rodentium* day 12). Representative plots of CD4^+^CD44^+^Ly6C^+^CD162^+^ T_eff_ cells and CD4^+^CD44^+^CXCR5^+^PD-1^+^Ly6C^−^CD162^−^ T_FH_ cells with histograms displaying Bcl-6 expression (**a**). Frequencies of T_FH_ cells in CD4^+^CD44^+^ gate (**b**) and the ratio of T_FH_:T_eff_ cells (LCMV *n* = 8, influenza *n* = 8, *T.* *muris* *n* = 6, *H.* *polygyrus* *n* = 9, *C.* *rodentium* *n* = 10 mice per group) (**c**). Representative plots of ZsGreen_T-bet reporter expression (T_FH_ cells are blue, T_eff_ cells are red) (**d**). ZsGreen_T-bet reporter^+^ frequency and gMFI of CD4^+^CD44^+^CXCR5^+^PD-1^+^Ly6C^−^CD162^−^ T_FH_ cells (LCMV *n* = 8, influenza *n* = 8, *T.* *muris* *n* = 6, *H.* *polygyrus* *n* = 10, *C.* *rodentium* *n* = 7 mice per group) (**e**). Immunofluorescence staining of draining lymph node GCs. Red arrows indicate ZsGreen_T-bet reporter^+^ T_FH_ cells. Yellow, CD4; blue, IgD; magenta, GL7; green, ZsGreen_T-bet reporter. Scale bar, 200 μm (**f**). Correlation of ZsGreen_T-bet reporter expression with the T_eff_:T_FH_ cell ratio across infections (**g**). Frequency of T_FH_ cells that produced IFNγ, IL-4 or IL-17A (**h**). IFNγ^+^ T_FH_1, IL-4^+^ T_FH_2, and IL-17^+^ T_FH_17 cells are included in the entire T_FH_ population (**i**). The inner slice displays cytokine coexpression. The outer ring (green) indicates the proportion of ZsGreen_T-bet^+^ T_FH_ cells expressed. The data are from 6–10 mice per group and are presented as the mean ± s.e.m. Statistical tests included one-way analysis of variance (ANOVA) for multiple comparisons and Pearson correlation with two-tailed *P* values. ****P < 0.0001 or otherwise indicated. Source Data

**Extended Data Fig. 1:**
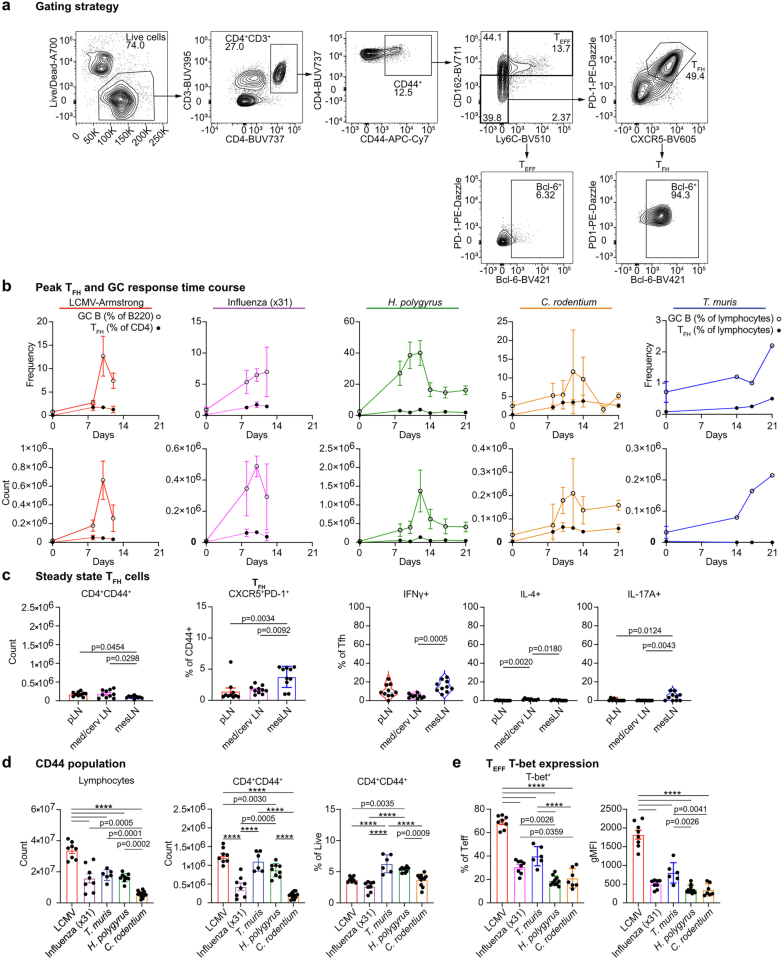
Induction of T_FH_ response following diverse infection compared to steady state homeostasis. **a**, Gating strategy to identify CD4^+^CD44^+^CXCR5^+^PD-1^+^Ly6C^−^CD162^−^ T_FH_ and CD4^+^CD44^+^Ly6C^+^CD162^+^ T_EFF_ cells and Bcl-6 expression. **b**, Timecourse analysis of draining lymph node GC cells from wild-type mice infected with indicated pathogens displaying frequencies and counts of CD4^+^CD44^+^CXCR5^+^PD-1^+^Ly6C^−^CD162^−^ T_FH_ cells (closed circle) and B220^+^IgD^lo^CD95^+^ GC B cells (open circle) (LCMV n = 3, influenza n = 4, *H. polygyrus* n = 4, *C. rodentium* n = 3 mice per group). *T.muris* data in^7^. **c**, Steady state analysis of indicated lymph nodes from uninfected wild-type mice. CD4^+^CD44^+^ cell counts, frequency of CD4^+^CD44^+^CXCR5^+^PD-1^+^Ly6C^−^CD162^−^ T_FH_ cells and frequency of T_FH_ cells produced IFNγ, IL-4, and IL-17 (n = 10 mice per group). **d**,**e** Analysis of draining lymph node cells from (**d**) wild-type and (**e**) ZsGreen_T-bet reporter mice infected with indicated pathogens at early peak GC response. (**d**) Total lymphocyte counts, CD4^+^CD44^+^ cell counts and frequency of CD4^+^CD44^+^ cells of live cells (LCMV n = 8, influenza n = 8, *T. muris* n = 6, *H. polygyrus* n = 9, *C. rodentium* n = 11 mice per group). (**e**) ZsGreen_T-bet reporter^+^ frequency and gMFI of CD4^+^CD44^+^Ly6C^+^CD162^+^ T_EFF_ cells (LCMV n = 8, influenza n = 8, *T. muris* n = 6, *H. polygyrus* n = 10, *C. rodentium* n = 7 mice per group). Data show experiments of 6–10 mice per group and mean ± SEM. Statistical tests: one-way ANOVA of multiple comparisons. **** P values are <0.0001 or otherwise indicated. Source Data

We hypothesized that pathogen-specific cues result in functionally distinct T_FH_ phenotypes with the capacity to tailor B cell responses. To test this, we investigated T_FH_ and T_eff_ differentiation and function during viral, helminth and bacterial infections. As T_FH_ cells evolve over time^11,33^, we assessed the early peak of T_FH_ and GC B cell accumulation for each infection, defined by T_FH_ and GC B cell frequency, in polyclonal T_FH_ (CD3^+^CD4^+^CD44^+^Ly6C^−^CD162^−^CXCR5^+^PD-1^+^Bcl-6^+^) and T_eff_ (CD3^+^CD4^+^CD44^+^Ly6C^+^CD162^+^CXCR5^−^PD-1^−^Bcl-6^−^) cell compartments after viral (acute Armstrong lymphocytic choriomeningitis virus (LCMV) or influenza A HKx31 H3N2 strain), helminth (*Trichuris* *muris*^7^ or *Heligmosomoides* *polygyrus*), and bacterial (*Citrobacter* *rodentium*) infections (Fig. 1a and Extended Data Fig. 1a,b). To maximize the influence of environmental cues, we analyzed the natural infection route and draining lymph nodes for each infection (inguinal, brachial and axillary lymph nodes for LCMV; mediastinal and cervical lymph nodes for influenza A; and mesenteric lymph nodes for the remaining pathogens) to investigate the breadth of T_FH_ phenotypes. T_FH_ cells were generated to varying degrees in all infections, as reflected by their frequency and the ratio between the T_FH_ and T_eff_ populations and compared to steady-state lymph nodes (Fig. 1b,c and Extended Data Fig. 1c,d).

**Extended Data Fig. 2:**
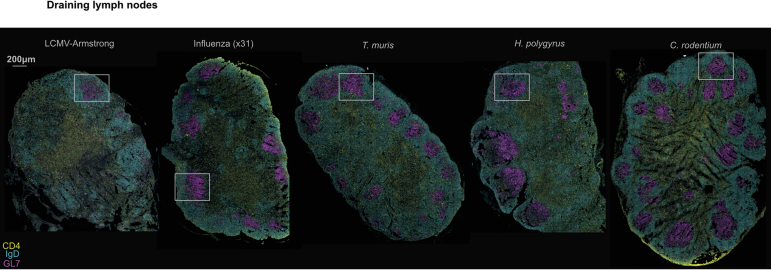
Conserved GC morphology in draining lymph nodes following infection with diverse pathogens. Immunofluorescent staining of draining lymph node of infected ZsGreen_T-bet reporter mice. Boxes indicate zoom of GC shown in Fig. 1f. Data are representative of 2–3 mice per group. Yellow: CD4, blue: IgD, magenta. Scale bar consistent for all images, 200μm. Source Data

The T_H_1 lineage-defining transcription factor T-bet (encoded by *Tbx21*) plays a context-specific role in T_FH_ bifurcation and function in response to viral infections^17,34^. We therefore sought to investigate the expression of the ZsGreen-T-bet reporter across diverse pathogen classes^35^. T_eff_ cells reported higher T-bet than T_FH_ cells in all infections (Fig. 1d,e and Extended Data Fig. 1e). The T-bet expression level was graded within the T_FH_ cell compartment (Fig. 1d,e). Confocal analysis revealed conserved draining lymph node and GC (GL7^+^IgD^−^) morphology (Extended Data Fig. 2). Within GC structures, we identified T-bet^+^ T_FH_ cells in LCMV-, influenza- and *T.* *muris*-infected tissue but not in *H.* *polygyrus* or *C.* *rodentium*, consistent with the increased level of T_FH_ T-bet expression in these infections (Fig. 1f). T-bet expression correlated with T_FH_:T_eff_ bifurcation (*r* = 0.7416), demonstrating that the context-specific role of T-bet extends beyond viral pathogens to helminth and bacterial infections (Fig. 1g)^7,19,34^.

**Extended Data Fig. 3:**
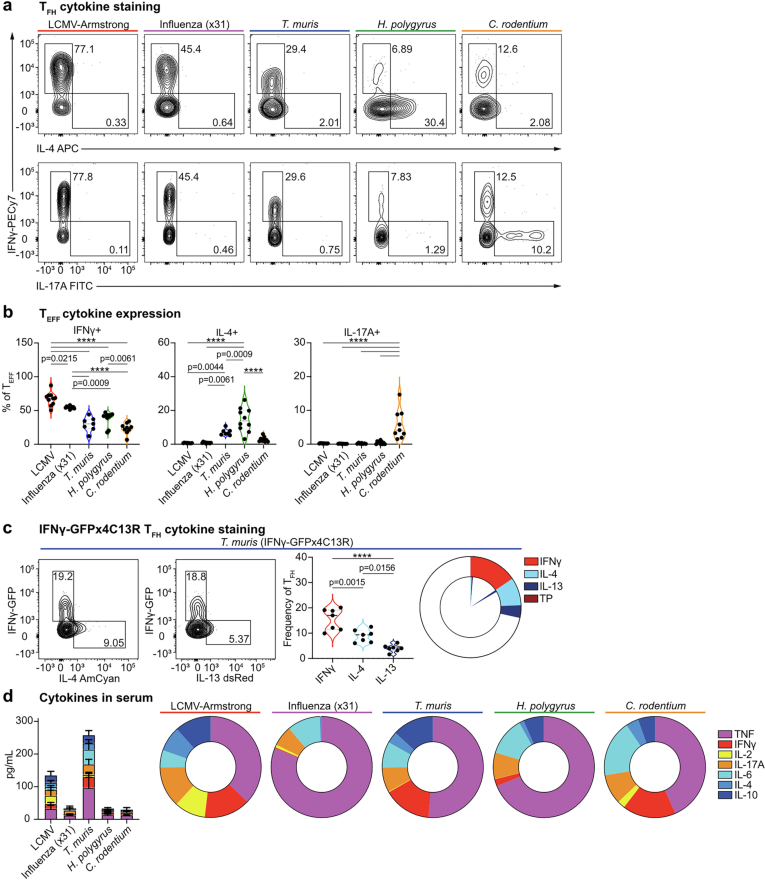
Heterogenous T_FH_ phenotypes display diverse cytokine profiles, similar to that of T_EFF_ and systemic cytokine milieu. **a–d** Analysis of (a–c) draining lymph node cells and (**d**) serum from (**a**,**b**,**d**) wild-type and (**c**) IFNγ-GFPx4C13R reporter mice infected with indicated pathogens at early peak GC response. (**a**) Representative plots of CD4^+^CD44^+^CXCR5^+^PD-1^+^Ly6C^−^CD162^−^ T_FH_ cell IFNγ, IL-4, and IL-17 cytokine production. (**b**) Frequency of CD4^+^CD44^+^Ly6C^+^CD162^+^ T_EFF_ cell produced IFNg, IL-4, IL-17A. (**c**) IFNγ-GFPx4C13R reporter expression in CD4^+^CD44^+^CXCR5^+^PD-1^+^Ly6C^−^CD162^−^ T_FH_ cells following *T. muris* infection. Representative plots, frequency of cytokine reporter with T_FH_ population and displayed as parts of whole T_FH_ population. Inner slice displays cytokine coexpression. (**d**) Serum cytokine concentration (pg/mL) in serum (n = 10 mice per group). Cytokines displayed as parts of total cytokines analyzed. Data show experiments of 7–10 mice per group and mean ± SEM. Statistical tests: one-way ANOVA of multiple comparisons. **** P values are <0.0001 or otherwise indicated. Source Data

As cytokine production defines T_FH_ cell function^15^, each pathogen-induced T_FH_ population displayed a distinct cytokine profile (Fig. 1h and Extended Data Fig. 3a). This mirrored T_eff_ cytokine production for each infection (Extended Data Fig. 3b). The majority of T_FH_ cells demonstrated cytokine specialization, with few cells producing IFNγ, IL-4 or IL-17 in combination (Fig. 1i, inner pie slice). Intracellular cytokine expression was confirmed via the use of IFNγ-GFPx4C13R reporter mice for *T.* *muris* infection which, in addition to similar IFNγ and IL-4 expression, indicated the presence of an IL-13-expressing T_FH_ population (Extended Data Fig. 3c). T_FH_ cytokine production largely reflected the serum cytokine milieu (Extended Data Fig. 3d). Furthermore, overlaying T_FH_ T-bet expression with T_FH_ cytokine production revealed that beyond the T_FH_:T_H_1 bifurcation, T-bet underlies the IFNγ^+^ T_FH_1 phenotype across all infections (Fig. 1i).

### T_FH_ phenotypes correlate with tailored B cell responses

**Fig. 2:**
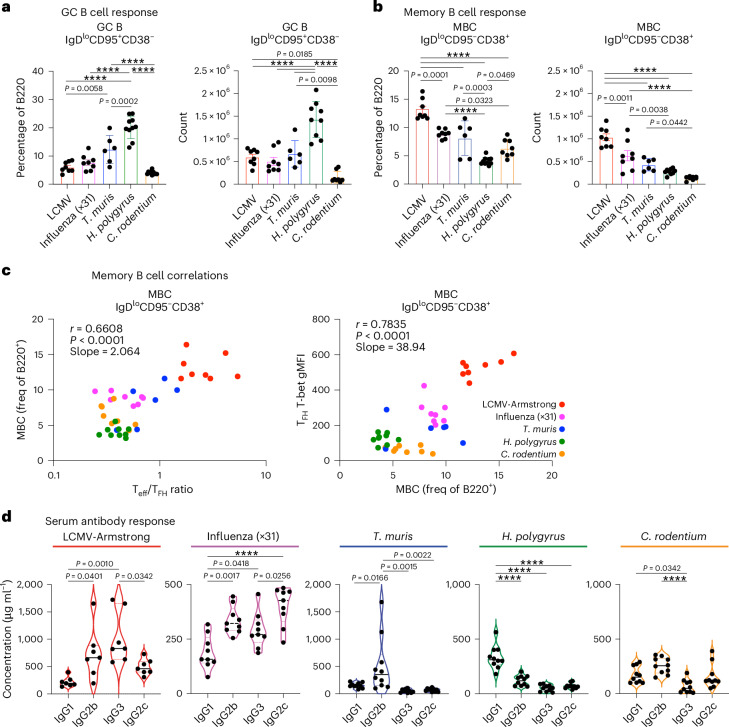
Diverse pathogen infection tailors B cell responses. **a**–**d**, Analysis of draining lymph node cells (**a**–**c**) and serum (**d**) from wild-type (**a**,**b**) and ZsGreen_T-bet (**c**) reporter mice infected with the indicated pathogens at the early peak GC response. Analysis of B220^+^IgD^lo^CD95^+^CD38^−^ GC B cells (**a**) and B220^+^IgD^lo^CD95^−^CD38^+^ MBCs (LCMV *n* = 8; influenza *n* = 8; *T.* *muris* *n* = 6; *H. polygyrus* *n* = 9; and *C.* *rodentium* *n* = 8 mice per group) (**b**). Correlation of the frequency of MBC with the ratio of T_eff_:T_FH_ cells and ZsGreen_T-bet reporter expression gMFI across infections (**c**). Serum IgG isotype concentration (**d**). The data are from 6–10 mice per group and are presented as the mean ± s.e.m. Statistical tests included one-way ANOVA for multiple comparisons and Pearson correlation with two-tailed *P* values. ****P < 0.0001 or otherwise indicated. Source Data

**Extended Data Fig. 4:**
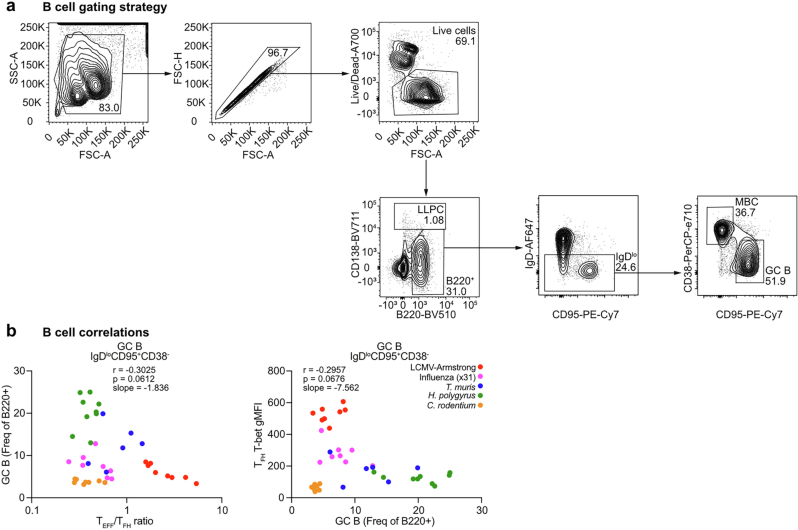
Pathogen-induced T_FH_ phenotypes correlate tailored B cell responses. **a**, Gating strategy to identify GC and MBC populations. **b**, Analysis of B220^+^IgD^lo^CD95^+^CD38^−^ GC B cells from ZsGreen_T-bet reporter mice infected with indicated pathogens at early peak GC response. (**b**) Correlation of frequency of GC B to the ratio of T_EFF_/T_FH_ cells and to ZsGreen_T-bet reporter expression gMFI across infections. Data show experiments of 6–10 mice per group. Statistical tests: Pearson correlation of two-tailed P value. Source Data

We next investigated how the variation in T_FH_ cytokine expression correlated with GC B cell and antibody production. GC B (B220^+^CD138^−^IgD^lo^CD38^−^CD95^+^) cells were quantified, with *H.* *polygyrus* generating the largest GC B cell response (Fig. 2a and Extended Data Fig. 4a). The frequency of GC B cells and the T_eff_:T_FH_ ratio were not statistically correlated across infections (*r* = −0.3025) (Extended Data Fig. 4b). In contrast, the relative frequency of memory B cells (MBCs; B220^+^CD138^−^IgD^lo^CD38^+^CD95^−^) correlated with a high T_eff_:T_FH_ ratio (*r* = 0.6608) as well as high T_FH_ T-bet^+^ expression (*r* = 0.7835) (Fig. 2b,c). Given that IL-4 progressively impacts MBC formation, these observed correlations may alter throughout GC formation and collapse^11^. Furthermore, serum antibody analysis demonstrated distinct IgG class-switch isotype expression, which was consistent with each infection producing a unique combination of T_FH_ cytokines (Fig. 2d). In combination, diverse pathogen infections induce distinct populations of T_FH_ cells that exhibit distinct cytokine profiles and correlate with GC and MBC output and with distinct antibody isotype usage.

### Core T_FH_ and T_FR_ transcriptional signatures

**Fig. 3:**
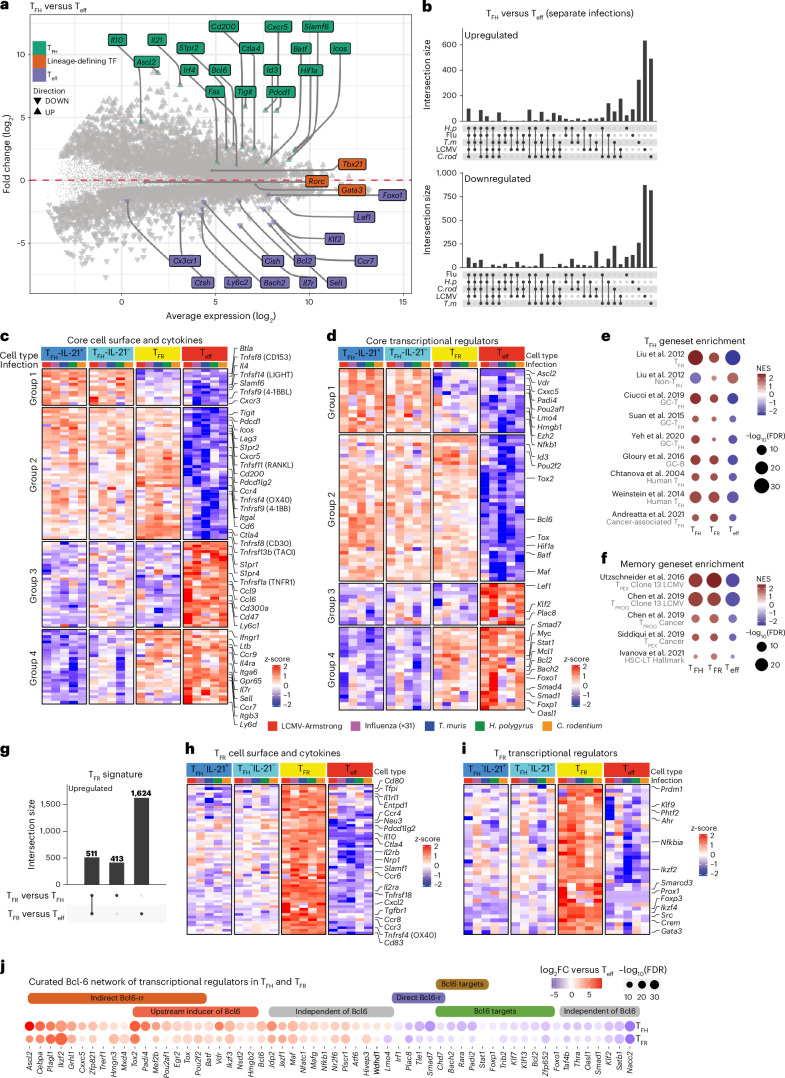
Identification of conserved T_FH_ and T_FR_ signatures. **a**–**j**, Bulk RNA-seq of sorted CD4^+^CD44^+^PD-1^+^CXCR5^+^FoxP3-RFP^−^IL-21–GFP^+^ T_FH_, CD4^+^CD44^+^PD-1^+^CXCR5^+^FoxP3-RFP^−^IL-21–GFP^−^ T_FH_, CD4^+^CD44^+^PD-1^+^CXCR5^+^FoxP3-RFP^+^IL-21–GFP^+^ T_FR_, and CD4^+^CD44^+^PD-1^−^CXCR5^−^FoxP3-RFP^−^IL-21–GFP^−^ T_eff_ cells from the draining lymph nodes of the mice infected with pathogens described in Fig. 1. MA plot visualizing the log fold change (FC) in the mean expression of genes expressed differentially between the T_FH_ and T_eff_ populations for the five infections (**a**). UpSet plot showing intersections of genes differentially expressed between the T_FH_ and T_eff_ populations for each infection (**b**). Common DE genes across three or more infections were used to derive the core T_FH_ signature. False discovery rate (FDR) < 0.05. Heatmap of core T_FH_ signature genes (row-based z scores of normalized log_2_ counts per million) for cytokine and cell surface receptor genes (**c**) and transcriptional regulator genes (**d**). GSEA of differentially expressed genes in T_FH_ (T_FH_ versus T_eff_ comparison), T_FR_ (T_FR_ versus T_eff_), and T_eff_ cells (T_eff_ versus T_FH_) for all infections (**e**,**f**). NES of T_FH_, non-T_FH_, GC T_FH_, GC B, human T_FH_ and cancer-associated T_FH_ cell programs (**e**) and precursors of exhausted (T_PEX_), exhausted progenitor (T_PROG_) and long-term hematopoietic stem cell (HSC-LT) gene programs in the T_FH_, T_FR_ and T_eff_ populations (**f**). The NES score represents the enrichment of genes (sets) relative to each comparison, correcting for multiple testing. UpSet plot of the T_FR_ signature showing intersections of upregulated genes expressed differentially between the T_FR_ versus T_FH_ cell contrast population and the T_FR_ versus T_eff_ cell contrast population for five infections (**g**). FDR < 0.05. Heatmap of T_FR_ signature genes shared across contrasts (T_FR_ versus T_FH_ and T_FR_ versus T_eff_) (row-based z score of normalized log_2_ counts per million) for cytokine and cell surface receptor genes (**h**) and transcriptional regulator genes (**i**). Bubble plot of Bcl-6 network transcriptional regulator genes in the T_FH_ core showing log_2_FC differences in the T_FH_ (T_FH_ versus T_eff_ contrast) and T_FR_ (T_FR_ versus T_eff_ contrast) populations (**j**). The size of the bubble represents the −log_10_(FDR), and the color indicates the log_2_FC compared to T_eff_ cells. The colored bar indicates genes present in the T_FH_ core and Bcl-6 gene sets from Extended Data Fig. 5g. Novel genes proposed to be independent of the Bcl-6 network are indicated with gray bars. The data represent independent samples of 2–3 per cell type per infection. Source Data

**Extended Data Fig. 5:**
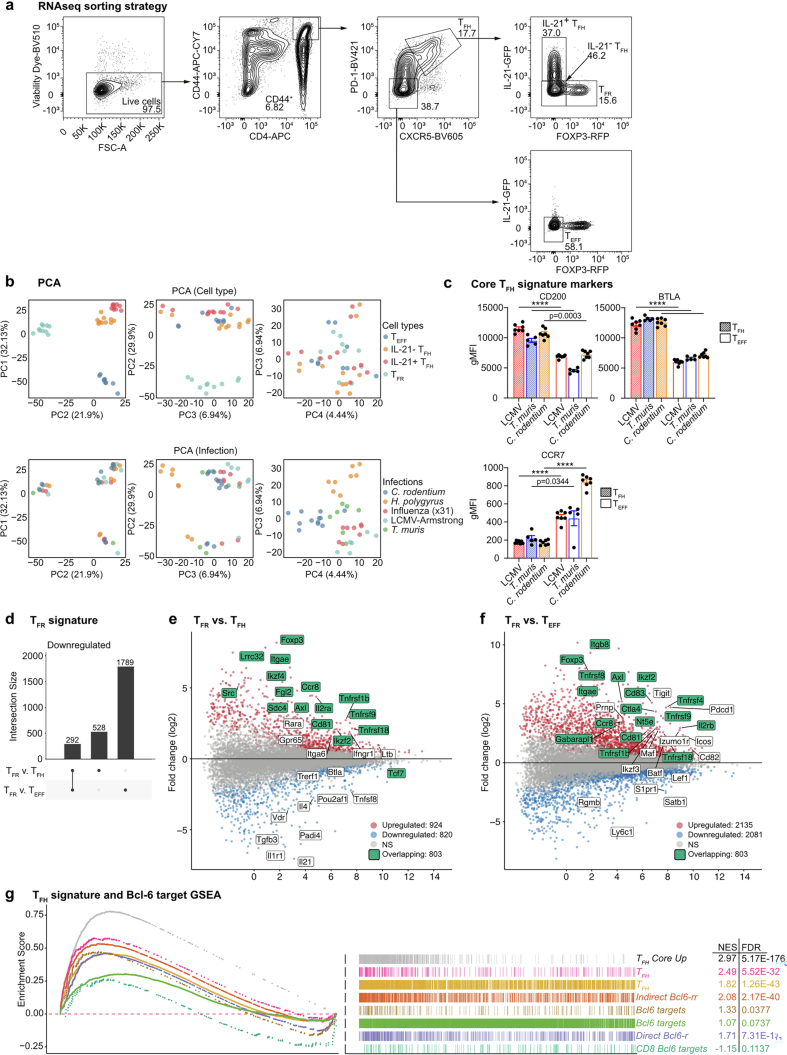
Pathogen-specific transcriptional programs separate by cell type and infection and establishment of T_FH_ and T_FR_ core signatures. RNA-seq of CD4^+^CD44^+^PD-1^+^CXCR5^+^FoxP3-RFP^−^IL-21–GFP^+^ T_FH_, CD4^+^CD44^+^PD-1^+^CXCR5^+^FoxP3-RFP^−^IL-21–GFP^−^ T_FH_, CD4^+^CD44^+^PD-1^+^CXCR5^+^FoxP3-RFP^+^IL-21–GFP^+^ T_FR_, and CD4^+^CD44^+^PD-1^−^CXCR5^−^FoxP3-RFP^−^IL-21–GFP^−^ T_EFF_ cells from draining lymph nodes of wild-type mice infected with indicated pathogens at early peak GC response. **a**, Gating strategy of sorted populations. **b**, Principal Component Analysis (PCA) plots of sorted IL-21^+^ T_FH_, IL-21^−^ T_FH_, T_FR_, and T_EFF_ cell transcriptomes stratified by either (top) cell type or (bottom) infection type. Data show independent samples of 2–3 per cell type per infection. **c**, Analysis of draining lymph node cells from wild-type mice infected with indicated pathogens. gMFI of upregulated (CD200 and BTLA) and downregulated (CCR7) core T_FH_ signature markers for CD4^+^CD44^+^CXCR5^+^PD-1^+^Ly6C^−^CD162^−^ T_FH_ cells. Data show experiments of 5–7 mice per group and mean ± SEM. **d**, UpSet plot of T_FR_ signature showing intersections of downregulated genes expressed differentially between T_FR_ vs. T_FH_ contrast and T_FR_ vs. T_EFF_ contrast for five infections. False discovery rate (FDR) < 0.05. **e**,**f**, MA plot visualizing log fold change against the mean expression of genes expressed differentially between (**e**) T_FR_ and T_FH_ cells and (**f**) T_FR_ and T_EFF_ cells for five infections. **g**, GSEA of differentially expressed genes in T_FH_ (T_FH_ vs. T_EFF_ contrast) and T_FR_ cells (T_FR_ vs. T_EFF_ contrast) combined for five infections. Barcode and ES plots displaying enrichment of genes (ES and NES) upregulated in core T_FH_ signature (T_FH_ Core Up), T_FH_ genes, genes indirectly regulated by Bcl-6 through repressor-of-repressor circuits (Indirect Bcl-6-rr), genes directly repressed by Bcl-6 (Direct Bcl-6-r), and Bcl-6 target genes (Bcl-6 targets) in T_FH_ cells (T_FH_ vs. T_EFF_) contrast^53,54,59,78,90^. Statistical tests: one-way ANOVA of multiple comparisons and Pearson correlation with two-tailed P value. **** P values are <0.0001 or otherwise indicated. Source Data

**Extended Data Fig. 6:**
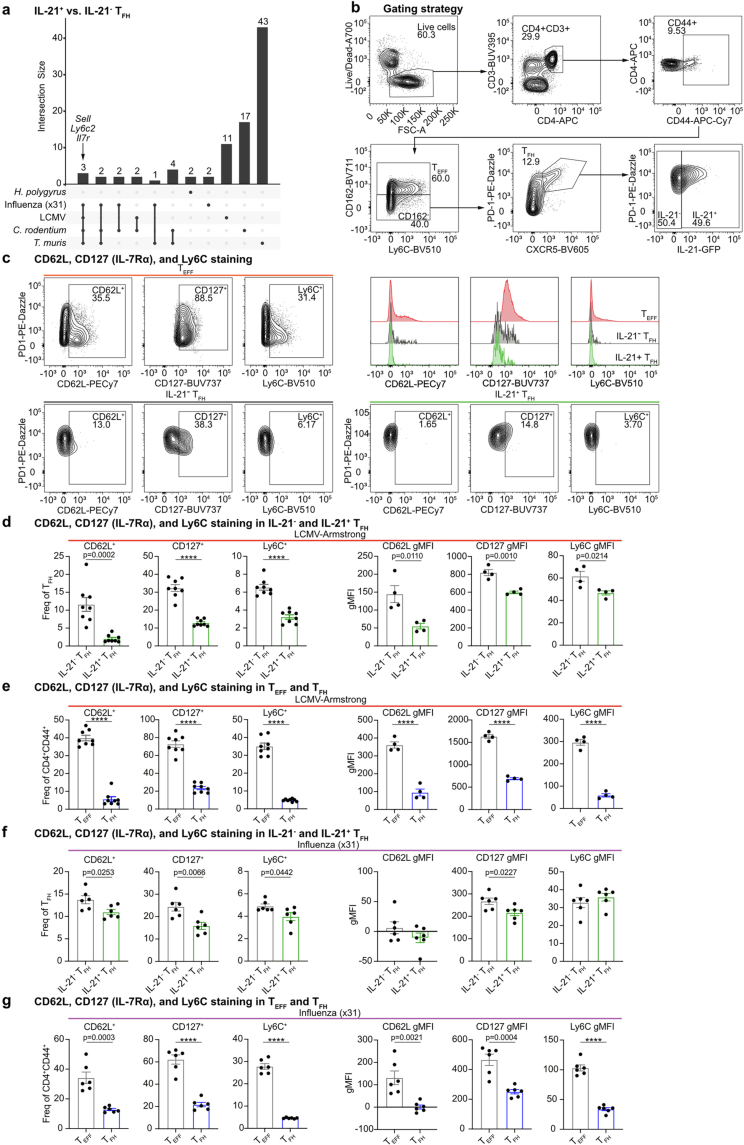
IL-21^+^ T_FH_ and IL-21^−^ T_FH_ cells are transcriptionally similar across diverse infections. **a**, UpSet plot displaying intersects of genes expressed differentially between IL-21^+^ T_FH_ and IL-21^−^ T_FH_ cells from separate infections in bulk RNA-seq analysis of CD4^+^CD44^+^PD-1^+^CXCR5^+^FoxP3-RFP^−^IL-21–GFP^+^ T_FH_ and CD4^+^CD44^+^PD-1^+^CXCR5^+^ FoxP3-RFP^−^IL-21–GFP^−^ T_FH_ cells as in Fig. 3. Three genes intersecting across 4 infections indicated with arrow. **b**-**g**, Analysis of CD4^+^CD44^+^PD-1^+^CXCR5^+^CD162^−^ T_FH_ cells and CD4^+^CD44^+^CD162^+^ T_EFF_ cells from draining lymph nodes from wild-type mice infected with indicated pathogens, LCMV or influenza A at early peak GC response. (**b**) Gating strategy to identify T_EFF_, IL-21^−^ T_FH_, and IL-21^+^ T_FH_ cells. (**c**) Representative plots and histograms of CD62L, CD127, and Ly6C staining on IL-21^−^ T_FH_, IL-21^+^ T_FH_ cells and T_EFF_ cells in LCMV infection. (**d**-**g**) Frequency and gMFI of CD62L^+^, CD127^+^, and Ly6C^+^ on (**d**,**f**) IL-21^−^ T_FH_, IL-21^+^ T_FH_ cells, frequency of total T_FH_ population (**e**,**g**) T_FH_ and T_EFF_, frequency of CD4^+^CD44^+^ population. (**d**-**e**) in LCMV infection (n = 8 mice per group), (**f**-**g**) in influenza A infection (n = 6 mice per group). Data show experiments of 4–6 mice per group and mean ± SEM. Statistical tests: Two-tailed unpaired Student’s *t* test. **** P values are <0.0001 or otherwise indicated. Source Data

We next performed RNA sequencing (RNA-seq) on GC CD4^+^ T cell populations to define a core T_FH_ transcriptional program that is centrally induced regardless of the pathogen or T_FH_ phenotype. For this, we analyzed T_FH_ (CD4^+^CD44^+^CXCR5^+^PD-1^+^) cells compared to T_eff_ (CD4^+^CD44^+^CXCR5^−^PD-1^−^) cells following LCMV, influenza, *T.* *muris*, *H.* *polygyrus* and *C.* *rodentium* infection (Extended Data Fig. 5a). As IL-21^+^ cells have been shown to exhibit increased GC residency (referred to as GC T_FH_) with distinct transcriptional profiles, we used the IL-21^GFP^ transcriptional reporter to profile both IL-21^+^ and IL-21^−^ T_FH_ cells^10,36^. Furthermore, we isolated T_FR_ (CD4^+^CD44^+^FoxP3^+^CXCR5^+^PD-1^+^) cells, a subset that represses GC size and facilitates the emergence of high-affinity GC B cells^37,38^. Principal-component analysis (PCA) revealed that all samples were separated by cell type on PC1 (32.13%) and PC2 (21.9%) (Extended Data Fig. 5b). IL-21^+^ T_FH_ and IL-21^−^ T_FH_ cells were transcriptionally distinct (Extended Data Fig. 6a); however, across infections, the intersection of similarly regulated genes revealed only *Ly6c2, Il7r* (encoding CD127), and *Sell* (encoding CD62L), were downregulated in the IL-21^+^ T_FH_ population in four of five infections (Extended Data Fig. 6a). Cell surface staining confirmed the regulation of these factors at the protein level in LCMV and influenza (Extended Data Fig. 6b–g). To define the core T_FH_ transcription program, we combined all T_FH_ samples and performed differential expression (DE) analysis relative to T_eff_ from each infection (Fig. 3a). Validating this approach, known T_FH_ genes (*Bcl6*, *Cxcr5*, *Pdcd1* (encoding PD-1) and *Cd200*) were upregulated, and known T_eff_ genes (*Klf2*, *Ccr7*, *Il7r*, *Ly6c1* and *Sell*) were downregulated (Fig. 3a). While *Tbx21*, *Gata3* and *Rorc* encode transcription factors that mediate the bifurcation between T_FH_ cells and their respective T_H_ subsets, the core T_FH_ signature was independent of these genes (Fig. 3a). In contrast, *Foxo1*, *Bach2* and *Foxp1* were present in the T_eff_ core signature, indicating their potential to act in opposition to the T_FH_ program regardless of the pathogen encountered^39–42^. We next performed pairwise DE analysis for each infection to establish a core T_FH_ signature that contained common significant directional regulation across three or more infections (608 upregulated and 635 downregulated genes) (Fig. 3b and Supplementary Table 1).

Analysis of genes encoding cell surface proteins, cytokine signaling components and transcription factors revealed that the core T_FH_ signature fell into four groups (1) T_FH_ core genes downregulated by both T_eff_ and T_FR_ cells; (2) T_FH_ genes coexpressed in T_FR_ cells; (3) T_eff_ core genes downregulated by T_FH_ and T_FR_ cells; and (4) T_eff_ genes coexpressed in T_FR_ cells (Fig. 3c,d) (Supplementary Table 1). Group 1 highlighted genes required for T_FH_ function and receptors that promote GC positioning and B cell interactions (*Btla* and *Il4*)^11,43^, genes that assist in the transition from T_FH_ to T_FR_ (*Sostdc1*)^44^, transcriptional and epigenetic regulators of cell fate (*Ascl2* and *Ezh2*)^9^ and genes not previously associated with T_FH_ identity or function (*Hmgb1*, *Padi4* and *Lmo4*). Group 2 contained genes required for T_FH_ and T_FR_ identity (*Bcl6*, *Cxcr5*, *Pdcd1*, *Id3*, *Icos*, *Cd200*, *Hif1a* and *Tnfrsf4* (encoding OX40)), along with genes suggestive of memory potential (*Tox*, *Tox2* and *Mxd4*)^45^. In contrast, the T_eff_ core signature (groups 3 and 4) included antagonists of T_FH_ lineage commitment (*Foxo1, Bach2* and *Foxp1*)^39–42^, T cell zone positioning receptors (*Ccr7*, *Sell* and *S1pr1*), inflammatory mediators and signaling (*Ly6c1*, *Ltb, Ifngr2*, *Oasl1* and *Smad7*) and proliferation and apoptosis inhibitors (*Mcl1*, *Bcl2* and *Myc*). Core upregulated (CD200 and BTLA) and downregulated (CCR7) cell surface molecules were confirmed at the protein level across multiple infections (Extended Data Fig. 5c). The core T_FH_ signature featured genes associated with GC T_FH_ cells (*Tigit*, *Maf* and *Plekho1*^)46^ (Fig. 3c,d). Furthermore, gene set enrichment analyses (GSEAs) with published datasets with normalized enrichment score (NES) confirmed that the T_FH_ cell core aligns with GC T_FH_ cells, GC B cells and additional infection-, vaccine- and cancer-induced T_FH_ cells in mice and humans (Fig. 3e). In line with the observation that both T_FH_ and T_FR_ cells (group 2 genes) expressed checkpoint receptors (*Pdcd1*, *Ctla4*, *Lag3* and *Tigit*) and the aforementioned drivers of long-term memory, GSEA-NES analysis revealed alignment with gene signatures of CD8^+^ T cell precursors and progenitors of exhausted cells from chronic infection and cancer and signatures associated with long-term hematopoietic stem cell memory relative to T_eff_ cells (Fig. 3f). Thus, the core T_FH_ signature is suggestive of increased memory potential, similar to that observed for stem-like memory CD8^+^ T cells^47^.

We next performed differential gene analysis to identify a T_FR_ cell transcriptional signature that was distinct from that of T_FH_ and T_eff_ cells (Fig. 3g and Extended Data Fig. 5d–f). This highlighted the effector status of T_FR_ cells coordinated by *Prdm1* (encoding BLIMP) and the expression of suppression genes (*Foxp3*, *Il10*, *Ctla4* and *Entpd1* (encoding CD39); Fig. 3h,i)^48,49^. Furthermore, this identified cytokine receptor (*Il2ra*, *Il2b* and *Il1rl1*) pathways, co-stimulation markers (*Tnfrsf18* (encoding GITR), *Tnfrsf4* (encoding OX40), *Cd80* and *CD83*) and chemokines and integrins that distinguish T_FR_ cells (Fig. 3h).

As Bcl-6 promotes multiple aspects of T_FH_ cell fate and function^9,50–52^, we next investigated how individual transcription factors expressed in both the T_FH_ and T_FR_ core signatures are influenced by Bcl-6. Aligned with the role of Bcl-6 as an obligate repressor, direct Bcl-6 targets were decreased in T_FH_ and T_FR_ cells relative to T_eff_ cells (Fig. 3j)^50,52–54^. Genes indirectly regulated by Bcl-6 through repressor-of-repressor (Bcl-6-rr) circuits showed increased expression in T_FH_ and T_FR_ cells (Fig. 3j and Extended Data Fig. 5g)^53^. Additionally, the Bcl-6-rr genes overlapped with genes known to be upstream inducers of Bcl-6. While a small group of transcriptional regulators were separate from known Bcl-6 circuits, the majority were within the Bcl-6 network, emphasizing that Bcl-6 underpins T_FH_ and T_FR_ differentiation independent of the pathogen class.

### Pathogen-specific T_FH_ transcriptional signatures

**Fig. 4:**
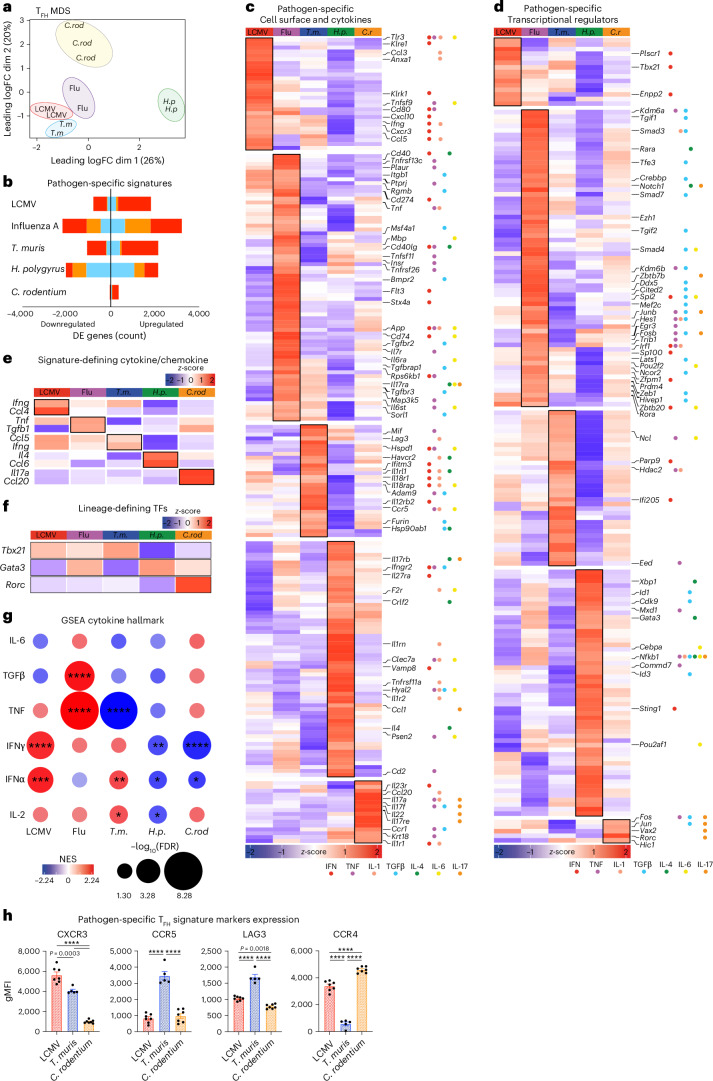
Identification of pathogen-specific T_FH_ phenotypes. **a**–**g**, Bulk RNA-seq of sorted CD4^+^CD44^+^PD-1^+^CXCR5^+^FoxP3-RFP^−^IL-21–GFP^+^ T_FH_, CD4^+^CD44^+^PD-1^+^CXCR5^+^FoxP3-RFP^−^IL-21–GFP^−^ T_FH_ cells from the draining lymph nodes of mice infected with the indicated pathogens (as in Fig. 1). MDS plot of CD4^+^CD44^+^PD-1^+^CXCR5^+^FoxP3-RFP^−^IL-21–GFP^+^ T_FH_ transcriptomes showing the separation of samples by infection type along dims 1 and 2 (**a**). Genes expressed differentially for T_FH_ (single infection vs. all other infections) and T_eff_ cells (single infection versus all other infections) (FDR < 0.05) (**b**). Orange, common pathogen-specific DE genes in both T_FH_ and T_eff_ cells; blue, pathogen-specific signatures unique to T_FH_ cells; red, pathogen-specific signatures unique to T_eff_ cells. Heatmaps of pathogen-specific T_FH_ signature genes (average row-based *z* score of normalized log_2_ counts per million) for selected cytokine and cell surface receptor genes (**c**) and transcriptional regulator genes (**d**). Cytokine pathway genes from Mouse Genome Informatic (MGI) database gene sets indicated in colored circles. IFN (red), TNF (purple), IL-1 (pink), TGFβ (blue), IL-4 (green), IL-6 (yellow) and IL-17 (orange). Heatmap of pathogen-specific T_FH_ signature genes (average row-based *z* score of normalized log_2_ counts per million) for selected signature-defining cytokines and chemokines for each infection (**e**) and lineage-defining transcription factors (**f**). GSEA of pathogen-specific signatures via MGI MSigDB ‘Hallmark’ gene sets for cytokine signaling and response (**g**). Bubble size represents the -log_10_(FDR), and color indicates the NES. The data represent independent samples of 2–3 per cell type per infection. **h**, Analysis of draining lymph node CD4^+^CD44^+^CXCR5^+^PD-1^+^Ly6C^−^CD162^−^ T_FH_ cells from wild-type mice infected with indicated pathogens (as in Fig. 1) displaying gMFI of pathogen-specific T_FH_ signature marker (LCMV *n* = 7; *T.* *muris* *n* = 5; and *C.* *rodentium* *n* = 7 mice per group). Data show experiments of 5–7 mice per group and mean ± s.e.m. Statistical tests were one-way ANOVA of multiple comparisons and Pearson correlation with two-tailed *P* values. ****P < 0.0001 or otherwise indicated. Source Data

While T-bet expression correlated with the number of IFNγ^+^ T_FH_1 cells (Fig. 1i), other regulators of T_FH_ heterogeneity are undefined. We therefore examined the pathogen-specific transcriptional programs of T_FH_ phenotypes. Multidimensional scaling (MDS) and PCA revealed that pathogen-directed T_FH_ cells were separated by infection into PC3 (6.94%) and PC4 (4.44%) (Fig. 4a and Extended Data Fig. 5b). DE analysis compared samples from each infection to the combined transcriptomes of all others for both the T_FH_ and T_eff_ populations. For influenza and *H.* *polygyrus*, a large proportion of pathogen-specific genes were shared between T_FH_ and T_eff_ cells, whereas this was not the case for LCMV or *T.* *muris* (Fig. 4b). We therefore defined pathogen-specific T_FH_ signatures to include the shared and T_FH_ distinct differentially regulated genes. Analysis of cell surface and cytokine genes revealed distinct cytokines and chemokines (*Ifng*, *Cxcl10*, *Il6*, *Ccl1*, *Il4*, *Il22* and *Il17*) and inhibitory and co-stimulatory molecules (*Cd80*, *Cd274* (encoding PD-L1), *Cd40* and *Havcr2* (encoding TIM3)) (Fig. 4c,e). Cell surface molecules (CXCR3, CCR5, LAG3 and CCR4) that indicated distinct pathogen-specific T_FH_ phenotypes were confirmed at the protein level across multiple infections (Fig. 4h). T_FH_ cells arising from each infection also expressed a distinct set of transcriptional and epigenetic regulators (Fig. 4d). We observed that *Tbx21*, *Gata3* and *Rorc* were differentially expressed within the pathogen-specific signatures (Fig. 4d,f). *Tbx21* and *Gata3* showed graded, reciprocal expression between infections, whereas *C.* *rodentium* was the only infection that induced T_FH_ cell *Rorc* expression (Fig. 4f).

### Cytokine signaling pathways instruct T_FH_ phenotypes

**Fig. 5:**
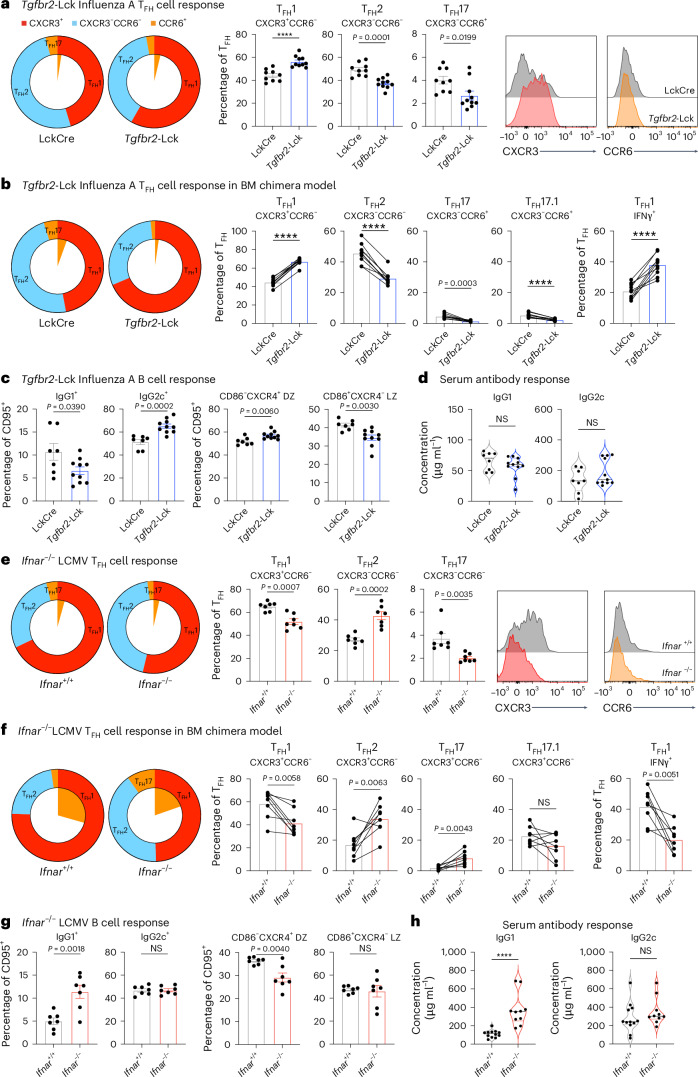
Pathogen-specific T_FH_ phenotypes are directed by distinct cytokine signaling pathways. **a**–**d**, Analysis of draining lymph nodes from *Tgfbr2*-Lck^Cre^ and Lck^Cre^ control mice infected with influenza A virus in intact mice (**a**,**c**,**d**) and 50:50 bone marrow chimera mice (**b**). CXCR3^+^ T_FH_1, CXCR3^−^CCR6^−^ T_FH_2, and CCR6^+^ T_FH_17 cells within CD4^+^CD44^+^CXCR5^+^PD-1^+^Ly6C^−^CD162^−^ T_FH_ cells are displayed as parts of the whole population (**a**,**b**). Frequency within T_FH_ population, and representative histograms of CXCR3 and CCR6 expression on T_FH_ cells (*n* = 9–10 mice per group) (**a**). The inner slice of the pie chart displays CXCR3^+^CCR6^+^ coexpression. Wild-type and *Tgfbr2*-Lck^Cre^ cells were identified by congenic marker expression (**b**). Frequency of CXCR3^+^ T_FH_1, CXCR3^−^CCR6^−^ T_FH_2, and CCR6^+^ T_FH_17 cells and frequency of IFNγ^+^ T_FH_1 cells within CD4^+^CD44^+^CXCR5^+^PD-1^+^Ly6C^−^CD162^−^ T_FH_ cells (*n* = 10 mice per group). The inner slice of the pie chart displays CXCR3^+^CCR6^+^ coexpression. IgG1 or IgG2c class switched B220^+^IgD^lo^CD95^+^ GC B cells and CD86^−^CXCR4^+^ dark zone (DZ) or CD86^+^CXCR4^−^ light zone (LZ) B cells on B220^+^IgD^lo^CD95^+^ GC B cells (*n* = 7–10 mice per group) (**c**). Serum IgG isotype concentration (**d**). **e**–**h** Draining lymph node analysis of *Ifnar*^−/−^ and *Ifnar* ^+/+^ control mice infected with LCMV in intact mice (**e**,**g**,**h**) and 50:50 bone marrow chimera mice (**f**). CXCR3^+^ T_FH_1, CXCR3^−^CCR6^−^ T_FH_2, and CCR6^+^ T_FH_17 cells within CD4^+^CD44^+^CXCR5^+^PD-1^+^Ly6C^−^CD162^−^ T_FH_ cells are displayed as parts of the whole population (**e**,**f**). Frequency within T_FH_ population, and representative histograms of CXCR3 and CCR6 expression on T_FH_ cells (*n* = 7 mice per group) (**e**). The inner slice of the pie chart displays CXCR3^+^CCR6^+^ coexpression. Wild-type and *Ifnar*^−/−^ cells were identified by congenic marker expression (**f**). Frequency of CXCR3^+^ T_FH_1, CXCR3^−^CCR6^−^ T_FH_2, and CCR6^+^ T_FH_17 cells and frequency of IFNγ^+^ T_FH_1 cells within CD4^+^CD44^+^CXCR5^+^PD-1^+^Ly6C^−^CD162^−^ T_FH_ cells (*n* = 8 mice per group). The inner slice of the pie chart displays CXCR3^+^CCR6^+^ coexpression. IgG1 or IgG2c class switched B220^+^IgD^lo^CD95^+^ GC B cells and CD86^−^CXCR4^+^ DZ or CD86^+^CXCR4^−^ LZ B cells from B220^+^IgD^lo^CD95^+^ GC B cells (*n* = 7 mice per group) (**g**). Serum IgG isotype concentration (**h**). The data are from 7–10 mice per group and are presented as the mean ± s.e.m. Statistical tests were a two-tailed unpaired Student’s *t*-test for the intact system and a two-tailed paired Student’s *t*-test for the bone marrow chimera model. ****P < 0.0001 or otherwise indicated. Source Data

**Extended Data Fig. 7:**
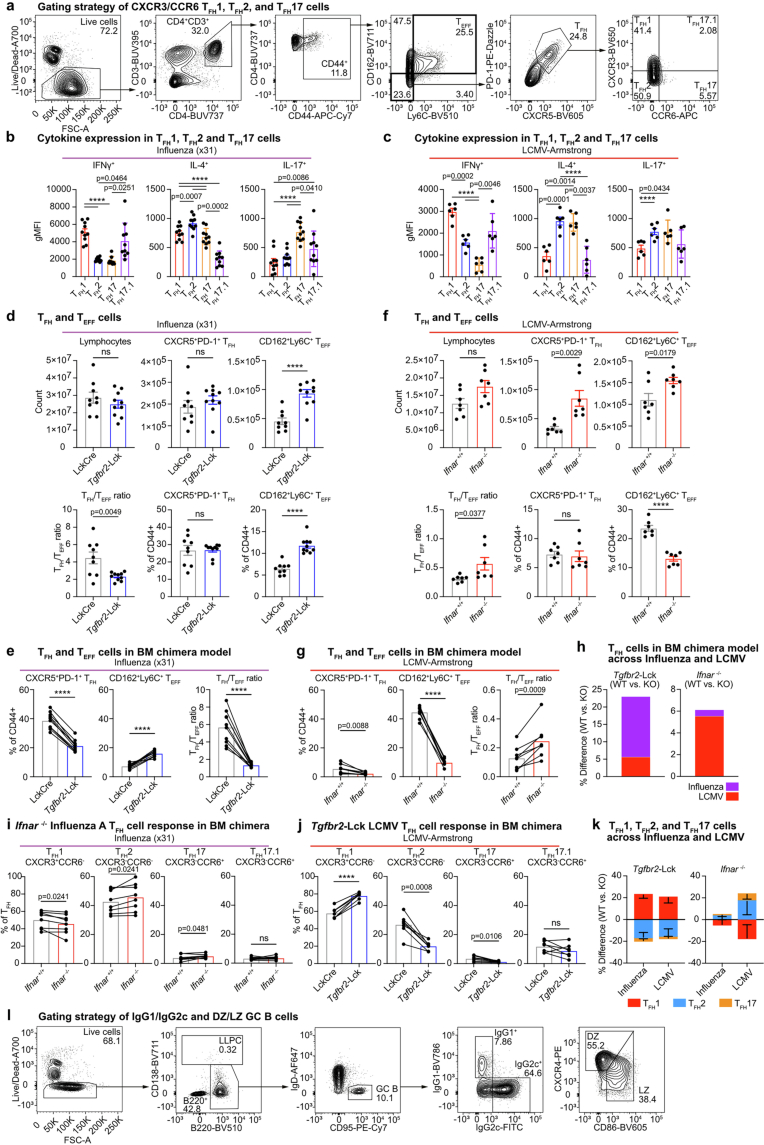
Pathogen-induced cytokine signaling influence T_FH_ and T_EFF_ bifurcation, T_FH_ subpopulations, and T_FH_ cells capacity to direct B cell and antibody output. **a**, Gating strategy to identify CD4^+^CD44^+^CXCR5^+^PD-1^+^Ly6C^−^CD162^−^ T_FH_1, T_FH_2, T_FH_17, and T_FH_17.1 phenotypes based on expression of CXCR3 and CCR6 chemokine receptors. **b**-**k**, Analysis of draining lymph nodes from (**b,c**) wild-type, (**d**) *Tgfbr2*-Lck^Cre^ and Lck^Cre^ mice, (**f**) *Ifnar*^−/−^ and *Ifnar*^+/+^ mice, (**e,h,j,k**) wild-type and *Tgfbr2*-Lck^Cre^ 50:50 bone marrow chimeras, or (**g-i,k**) wild-type and *Ifnar*^−/−^ 50:50 bone marrow chimera mice infected with (**b,d,e,h,i,k**) influenza or (**c**,**f,g,h,j,k**) LCMV. (**b**,**c**) gMFI expression of IFNg, IL-4, and IL-17 in T_FH_1 (CXCR3^+^CCR6^−^), T_FH_2 (CXCR3^−^CCR6^−^), T_FH_17 (CXCR3^−^CCR6^+^) and T_FH_17.1 (CXCR3^+^CCR6^+^) populations (influenza n = 10, LCMV n = 6 mice per group). (**d**,**f**) T_FH_ and T_EFF_ cell counts, T_FH_ and T_EFF_ cell frequencies, and T_FH_/T_EFF_ cell ratio (influenza n = 9-10, LCMV n = 7 mice per group). (**e**,**g**) T_FH_ and T_EFF_ cell frequencies and T_FH_/T_EFF_ cell ratio (influenza n = 10, LCMV n = 8 mice per group). (**h**) Comparison of effect of *Tgfbr2*-Lck^Cre^ and *Ifnar* ^−/−^ on T_FH_ frequency in chimeras. (**i**-**j**) Frequency of T_FH_1, T_FH_2, T_FH_17 and T_FH_17.1 cells within T_FH_ population in chimeric mice (influenza n = 8, LCMV n = 7 mice per group). (**k**) Comparison of effect of *Tgfbr2*-Lck^Cre^ and *Ifnar* ^−/−^ on T_FH_1, T_FH_2 and T_FH_17 cell frequency in influenza and LCMV infection in chimeras (*Tgfbr2*-Lck^Cre^ influenza n = 10, *Tgfbr2*-Lck^Cre^ LCMV n = 7, *Ifnar* ^−/−^ influenza n = 8, *Ifnar* ^−/−^ LCMV n = 8 mice per group). **l**, Gating strategy to identify IgG1 and IgG2 class switched GC B cells, and dark zone (DZ) and light zone (LZ) GC B cells. Data show experiments of 6–10 mice per group and mean ± SEM. Statistical tests: Two-tailed unpaired Student’s *t* test for intact system; two-tailed paired Student’s *t* test for bone marrow chimera model. **** P values are <0.0001 or otherwise indicated. Source Data

Pathogen-specific T_FH_ signatures indicated multiple genes that aligned to distinct cytokine signaling pathways (Fig. 4c,d). Therefore, we performed GSEA on the cytokine hallmark gene sets of the MSigDB database to understand the cytokine signaling pathways that may direct different T_FH_ phenotypes. Type I and type II IFN (IFN-I and IFN-II, respectively) signaling genes were positively enriched in T_FH_ cells from LCMV and *T.* *muris* (Fig. 4g). In contrast, these pathway genes were negatively enriched for *H.* *polygyrus* and *C.* *rodentium*, indicating IFNs as drivers of pathogen-specific T_FH_ phenotypes. Notably, despite being a viral infection the cytokine signaling observed in T_FH_ cells from influenza was distinct from those in LCMV-stimulated cells (Fig. 4c,d,g). Instead, T_FH_ cells in influenza were enriched for TGFβ signaling. These data support the concept that cytokine pathways are a stronger driver of specific heterogeneous T_FH_ cell phenotypes over pathogen class (Fig. 4c–e,g). We next investigated the role of TGFβ and IFN-I signaling in infection models dominated by the cytokine pathways of influenza and LCMV, respectively. For this purpose, the T_FH_ phenotypes were categorized into T_FH_1, T_FH_2 and T_FH_17 cell subpopulations according to CXCR3 and CCR6 chemokine expression, which aligns with differential cytokine production (Extended Data Fig. 7a–c). Consistent with previous work, targeted T cell deletion of TGFβR2 (*Tgfbr2*^fl/fl^Cre^LCK^) decreased the T_FH_:T_eff_ cell ratio (Extended Data Fig. 7d). In addition, the T_FH_ cell phenotype composition was altered, with increased frequency of T_FH_1 and decreased T_FH_2 and T_FH_17 cell populations (Fig. 5a). Mixed (50:50 wild-type:*Tgfbr2*^fl/fl^Cre^LCK^) bone marrow chimeras were generated to demonstrate that TGFβ modified the T_FH_ cell phenotype in a cell-specific manner (Fig. 5b and Extended Data Fig. 7e). An altered T_FH_ cell phenotype impacted the B cell response, with decreased frequency of cell surface IgG1^+^ and reciprocally increased IgG2c^+^ GC B cells (Fig. 5c,d). Additionally, GC cycling was dysregulated (Fig. 5c and Extended Data Fig. 7l). IFNAR deficiency (*Ifnar*^−/−^) resulted in reciprocal alterations in T_FH_:T_eff_ generation compared to TGFβR2 deficiency (Extended Data Fig. 7f). Within T_FH_ cells, the loss of IFN-I signaling decreased the frequency of T_FH_1 and T_FH_17 cells and increased the T_FH_2 cell phenotype (Fig. 5e). Mixed (50:50 wild-type:*Ifnar*^−/−^) bone marrow chimeras confirmed that the altered T_FH_ phenotypes were cell intrinsic (Fig. 5f and Extended Data Fig. 7g). Furthermore, these altered T_FH_ cell phenotypes were reflected in the B cell response, with increased IgG1^+^ GC B cells, decreased dark zone GC B cells and increased serum IgG1 concentrations (Fig. 5g,h). To investigate how context-specific cytokine signals drive T_FH_ phenotypes, we tested each mixed chimera with the alternative infection. This demonstrated that both TGFβ and IFN-I mediate the context-dependent regulation of T_FH_:T_eff_ cell bifurcation (Extended Data Fig. 7h). TGFβR2 deficiency altered T_FH_ cell composition in both influenza and LCMV infection (Extended Data Fig. 7i–k). In contrast, IFN-I had a more dominant role for T_FH_ composition in LCMV indicating it is a pathogen-specific factor that alters T_FH_ phenotype, function and B cell outcomes. Combined, this provides new insight into how cytokine signaling networks integrate to shape the functional T_FH_ cell response to direct B cell and GC output.

### Heterogeneous T_FH_ phenotypes in human tissue

**Fig. 6:**
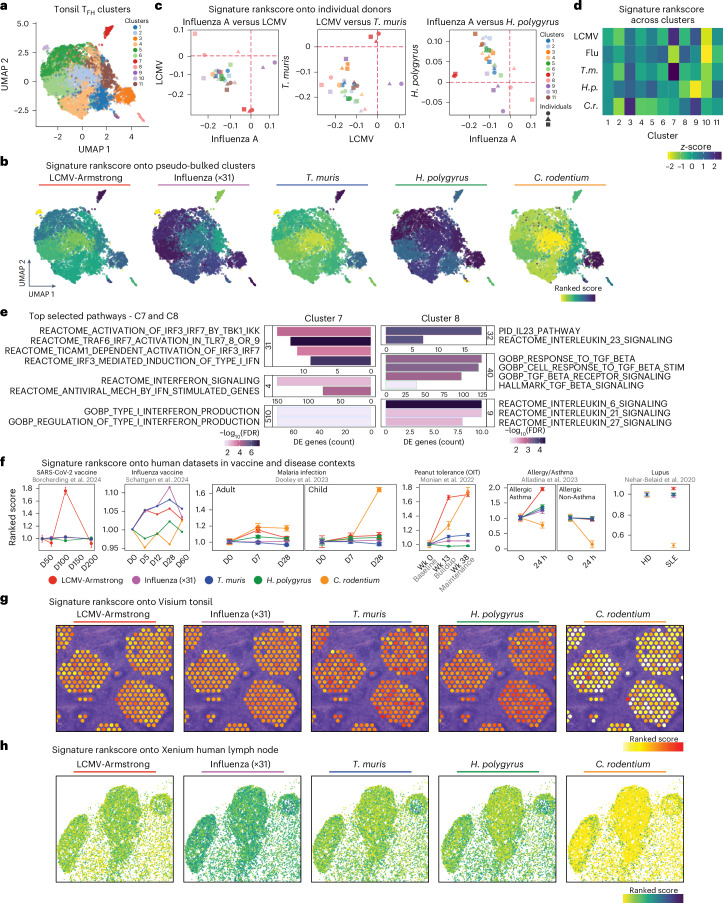
Cytokine-guided T_FH_ phenotypes are evident in human lymphoid tissue. **a**–**e**, scRNA-seq of sorted CD3^+^CD4^+^CD45RA^−^CD45RO^+^CXCR5^+^CD27^+^ human tonsillar T_FH_ cells from three healthy adult donors. UMAP dimensional reduction of data depicting 11 clusters on the basis of Louvain clustering via the Jaccard similarity index (*k* = 9) (**a**). Overlay of the mean ranked scores of each pathogen-specific signature onto UMAP clusters (**b**). Mean of the ‘TotalScore’ from the singscore: simpleScore function collated for each cluster. Pathogen-specific upregulated signature genes used. Rank scores of pathogen-specific signatures for each pseudosample on the basis of cluster and individual donors (**c**). Influenza A versus LCMV (left); LCMV versus *T.* *muris* (middle); influenza A versus *H.* *polygyrus* (right). Heatmap of the mean rank scores of pathogen-specific signatures for each cluster (row-based *z* score of the mean rank score) (**d**). GSEA-vissE analysis for the comparison of C7 versus C8 (**e**). Top gene sets of selected clusters as bar plots of DE gene counts with corresponding gene statistics (FDR as color shade) from the DE analysis. GSEA was performed via the Hallmarks c2 (‘CP:REACTOME’, ‘CP:PID’, ‘CP:BIOCARTA’,‘CP:KEGG’) and c5 (‘GO:BP’,‘GO:MF’) collections from the MSigDB using a two-tailed approach correcting for multiple testing with the FDR adjusted to *P* ≤ 0.05. **f**, Ranked score of pathogen-specific signatures in human dataset T_FH_ cells from donors in SARS-CoV-2 vaccine (*n* = 5), influenza vaccine (*n* = 1), malaria infection (adult *n* = 3, child *n* = 3), peanut allergy (*n* = 3), asthma (*n* = 4) and autoimmune contexts (healthy donor *n* = 6, systemic lupus erythematosus *n* = 8). Ranked scores normalized to the first time point for signatures. Data are presented as mean ± s.e.m. **g**, Visium spatial tonsil data displaying a ranked score of pathogen-specific signatures onto GCs highlighted by the core T_FH_ cell signature. **h**, Xenium spatial human lymph node data displaying a ranked score of pathogen-specific signatures in T_FH_ cells and GCs highlighted by the core T_FH_ cell signature. Source Data

**Extended Data Fig. 8:**
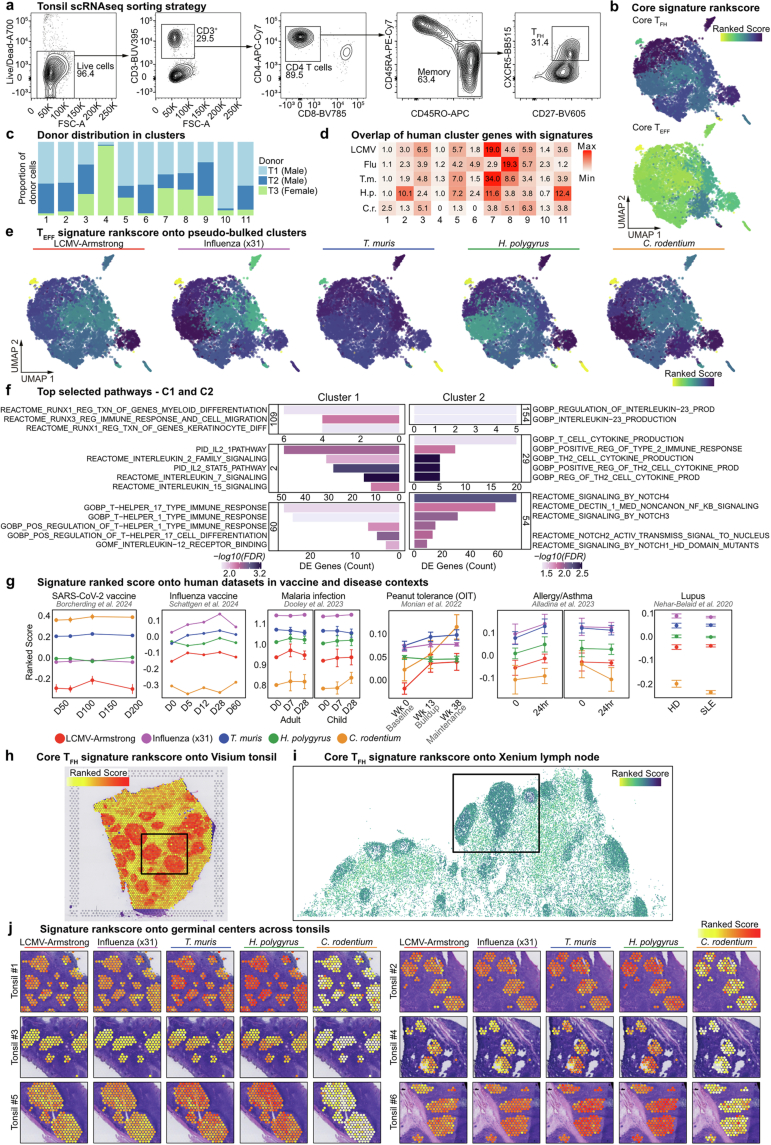
Human lymphoid tissue T_FH_ phenotypes identified by core and pathogen-specific T_FH_ signatures. **a**, Gating strategy to sort CD3^+^CD4^+^CD45RA^−^CD45RO^+^CXCR5^+^CD27^+^ human tonsillar T_FH_ cells from three healthy adult donors for paired scRNA-seq and CITE-seq. **b**, Overlay of mean ranked scores of core T_FH_ and T_EFF_ core signatures onto UMAP clusters. Mean of the ‘*TotalScore’* from *singscore::simpleScore* function collated for each cluster. **c**, Distribution of T_FH_ cells across 11 clusters by donor. **d**, Proportion of pathogen-specific T_FH_ signatures overlapping with human cluster genes expressed differentially (one cluster vs. all other clusters). **e**, Overlay of mean ranked scores of each T_EFF_ cell pathogen-specific signature onto UMAP clusters. Mean of the ‘*TotalScore’* from *singscore::simpleScore* function collated for each cluster. T_EFF_ cell pathogen-specific upregulated signature genes used. **f**, Gene set Enrichment and vissE analyses for the comparison Cluster 1 vs. Cluster 2. Top gene sets clusters of interest shown as barplot of DE gene counts with corresponding gene statistics (FDR as color shade) from the DE analysis. GSEA was performed using Hallmarks c2 (‘CP:REACTOME’, ‘CP:PID’, ‘CP:BIOCARTA’,‘CP:KEGG’) and c5 (‘GO:BP’,‘GO:MF’) collections from the MsigDB using a two-tailed approach correcting for multiple testing with false discovery rate (FDR) adjusted *p* ≤ 0.05. **g**, Ranked score of pathogen-specific signatures onto human dataset T_FH_ cells from donors in SARS-CoV-2 vaccine (n = 5), influenza vaccine (n = 1), malaria infection (adult n = 3, child n = 3), peanut allergy (n = 3), asthma (n = 4) and autoimmune contexts (healthy donor n = 6, systemic lupus erythematosus n = 8). Data are presented as mean values ± SEMs. **h**, Visium spatial tonsil data^113^ displaying ranked score of core T_FH_ signature. **i**, Xenium spatial human lymph node data displaying ranked score of core T_FH_ signature. **j**, Visium spatial tonsil data^113^ displaying ranked score of pathogen-specific signatures onto germinal centers highlighted by core T_FH_ signature. Source Data

Human tonsils are secondary lymphoid organs that are constantly exposed to the upper respiratory tract. Despite this, minimal tonsillar T_FH_ heterogeneity exists due to a lack of both transcriptional heterogeneity and identification of CXCR3^+^ and CCR6^+^ populations^9,55^. We hypothesized that our derived pathogen-specific T_FH_ signatures may provide new insight into T_FH_ heterogeneity within human lymphoid tissue. We performed paired single-cell RNA-seq (scRNA-seq) and surface protein sequencing (CITE-seq) analysis on T_FH_ cells (CD3^+^CD4^+^CD45RA^−^CD45RO^+^CXCR5^+^) from tonsils of adults with sleep apnea but otherwise ostensibly healthy individuals (Extended Data Fig. 8a,b and Supplementary Table 2; Donor Table 1). Unsupervised Louvain clustering of 24,393 T_FH_ cells identified 11 transcriptionally distinct T_FH_ cell clusters (C) (Fig. 6a). C4 disproportionally consisted of cells from the female donor; however, removing X-chromosome genes from the dataset had minimal effect on cluster distribution and DE gene analysis (Extended Data Fig. 8c and Supplementary Table 3). The ranked scores for pathogen-specific signatures (identified in Fig. 4) were overlaid onto Uniform Manifold Approximation and Projection (UMAP) pseudobulked clusters to indicate the proportion of pathogen-driven T_FH_ cell signatures evident within human T_FH_ cell clusters (Fig. 6b and Extended Data Fig. 8d). Overlap between mouse and human datasets was considerable considering the experimental differences in acquiring cells from acute pathogen infection and healthy human lymphoid tissue (Extended Data Fig. 8d). Further, the core T_eff_ (T_FH_ downregulated) cell signature was not evident in our human T_FH_ cell dataset (Extended Data Fig. 8b). Consistent with the overall cytokine milieu directing signatures, T_eff_ cell pathogen-specific signatures were present on the T_FH_ cell dataset, although these were less aligned with distinct human T_FH_ cell clusters (Extended Data Fig. 8e). For all three individual tonsil samples, the LCMV and influenza signatures aligned to C7 and C8, respectively (Fig. 6b–d). In addition to LCMV, *T.* *muris* also scored high on C7, whereas the *H.* *polygyrus* and *C.* *rodentium* signatures highlighted C2, C5 and C11, and C3 and C9, respectively (Fig. 6b–d). Dichotomy between clusters was observed, with inverse alignment between C8 and C7 when ranked on the basis of the influenza and LCMV or *T.* *muris* signatures, with C2 and C7 when ranked on the basis of *H.* *polygrus* and *T.* *muris*, and with C9 and C11 when ranked on the basis of the *C.* *rodentium and H.* *polygyrus* signatures (Fig. 6b,d). As C7 and C8 were the most separated from other T_FH_ populations and were the most polarized by pathogen-specific signatures, we performed GSEA and visualized the results via vissE analysis. Consistent with cytokine-guided differentiation, C7 was enriched in gene networks related to IFN signaling, whereas C8 was enriched in gene networks related to TGFβ, IL-23, IL-6, IL-21 and IL-27 signaling (Fig. 6e). Similarly, GSEA-vissE analysis of C1 revealed enrichment of cytokine signaling gene networks for T_H_17 cell and IL-2 family signaling responses, whereas C2 was enriched in T_H_2 cell pathway genes (Extended Data Fig. 8f). Thus, distinct cytokine pathways are evident in both mouse and human T_FH_ cell phenotypes.

To test the utility of cytokine-directed pathogen-induced T_FH_ transcriptional signatures, signatures were overlayed onto existing human datasets via a ranked score approach. Ranking context-specific transcriptional signatures onto longitudinal vaccine fine-needle aspirate scRNA-seq data following either SARS-CoV-2 or influenza vaccination demonstrated that distinct T_FH_ phenotypes dominate each response (Fig. 6f and Extended Data Fig. 8g). Furthermore, distinct T_FH_ phenotypes dominated cT_FH_ responses during adult and child malaria infection, during oral exposure to food antigen-tolerant immunotherapy, during allergy challenge, or in lupus patients compared to those in healthy donors (Fig. 6f and Extended Data Fig. 8g). Across these distinct immune-modifying conditions, the induction of the LCMV T_FH_ phenotype dominated in highly inflammatory settings (such as SARS-CoV-2 vaccination, allergic asthma and lupus), whereas the *C.* *rodentium* T_FH_ cell phenotype emerged in malaria infection in children and oral tolerance therapy and was specifically lost in non-allergic asthma and lupus disease (Fig. 6f).

Visium human tonsil atlas and publicly available Xenium steady-state human lymph node data showed that T_FH_ cell signatures were shared within GC structures (Fig. 6g,h and Extended Data Fig. 8h,i). While GCs within each tissue biopsy sample highlighted similar T_FH_ cell signature rankings, heterogeneity was observed when tonsillar tissue from different donors was compared (Extended Data Fig. 8j). These data suggest that cells exhibiting distinct T_FH_ cell phenotypes are capable of mixing within GC structures, but lymphoid tissue displays a dominant T_FH_ cell phenotype. Thus, identified T_FH_ cell signatures can be used to probe T_FH_ cell function following immune challenge or in antibody-mediated diseases.

### Cell surface identification of T_FH_ phenotypes

**Fig. 7:**
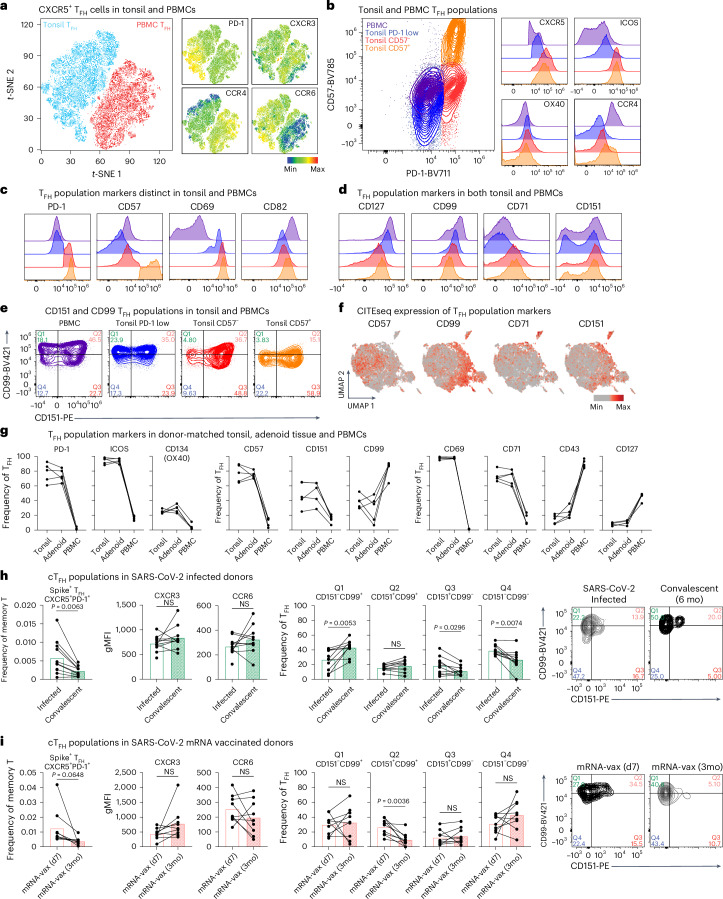
Cell surface proteins identify distinct human tonsillar T_FH_ and cT_FH_ phenotypes. **a**–**e**, Flow cytometry analysis of CD3^+^CD4^+^CD45RA^−^CD45RO^+^CXCR5^+^ T_FH_ cells from five PBMC and six tonsil healthy adult donors. *t*-distributed stochastic neighbor embedding (*t*-SNE) and heatmap expression of T_FH_ cell markers of PD-1, CXCR3, CCR6 and CCR4 (**a**). T_FH_ cell populations identified by PD-1 and CD57 with expression of the T_FH_ cell markers CXCR5, ICOS, OX40 and CCR4 on each population (**b**). Histograms of PD-1, CD57, CD69 and CD82 expression on T_FH_ cells (**c**). Histograms of CD127, CD99, CD71 and CD151 expression on T_FH_ cells (**d**). T_FH_ populations separated into quadrants (Q1–Q4) based on expression of CD99 and CD151 markers (**e**). **f**, scCITEseq surface protein expression (log counts) of sorted CD3^+^CD4^+^CD45RA^−^CD45RO^+^CXCR5^+^CD27^+^ human tonsillar T_FH_ cells from three healthy adult donors (as in **a**–**e**) of cluster-identifying markers overlaid onto the scRNA-seq UMAP. **g**, T_FH_ cell surface and activation markers in CD3^+^CD4^+^CD45RA^−^CD45RO^+^CXCR5^+^ human T_FH_ cells from matched tonsil, adenoid tissue and PBMC from five healthy juvenile donors. **h**,**i**, SARS-CoV-2 Spike tetramer^+^ CD3^+^CD4^+^CD45RA^−^CXCR5^+^ cT_FH_ cells from SARS-CoV-2 infected donors (**h**) at time of infection (11 of 11 donors; *n* = 11) and 6 months convalescence (10 of 11 donors; *n* = 10) and nine COVID-19 mRNA vaccinated donors (*n* = 9) (**i**) at 7 days post-vaccine and 3 months post-vaccine. Frequency of Spike tetramer^+^PD-1^+^ T_FH_ cells, gMFI expression for CXCR3 and CCR6, and frequencies and dot plots of CD151/CD99 quadrant-separated T_FH_ cell populations. Data show experiments of 8–11 samples per group. Statistical test was a two-tailed paired Student’s *t*-test. *P* values are indicated. Source Data

**Extended Data Fig. 9:**
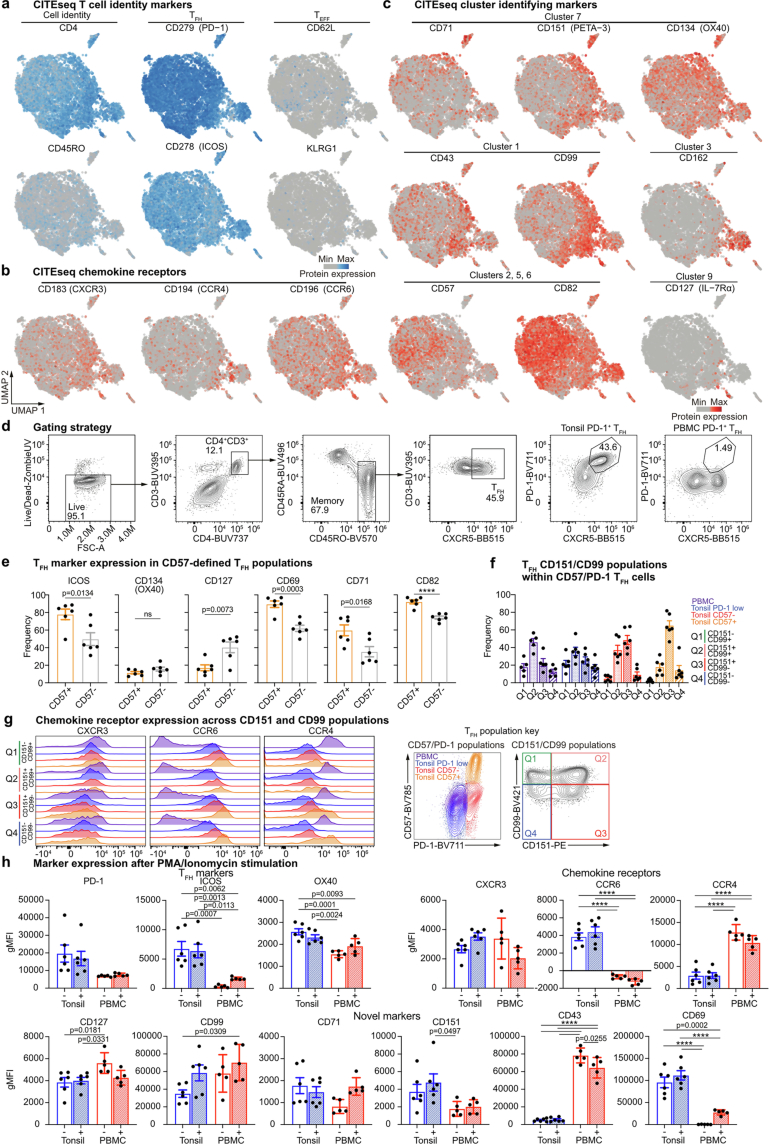
Novel markers identify distinct human T_FH_ subpopulations across tonsil and PBMC. a–c, scCITEseq surface protein expression of sorted CD3^+^CD4^+^CD45RA^−^CD45RO^+^CXCR5^+^CD27^+^ human tonsillar T_FH_ cells from three healthy adult donors (as in Fig. 7). scCITEseq surface protein expression (log counts) of (**a**) T cell identity markers, (**b**) chemokine receptors, and (**c**) cluster-identifying markers, overlaid onto scRNA-seq UMAP. **d**-**h**, CD3^+^CD4^+^CD45RA^−^CD45RO^+^CXCR5^+^ human T_FH_ cells from five PBMC samples and six tonsil samples from healthy adult donors. (**d**), Gating strategy to identify T_FH_ cells in human tonsil, adenoid, and PBMCs with PD-1^+^ expression in tonsil and PBMCs displayed in dot plots. (**e**) T_FH_ surface marker frequency in tonsil CD57^+^ or CD57^−^ T_FH_ cells (n = 6 samples per group). (**f**) CD151/CD99 T_FH_ populations (Q1-Q4) in T_FH_ cells distinguished by CD57 and PD-1 from tonsil (n = 6 tonsils per group) and PBMCs (n = 5 PBMCs per group). (**g**) CXCR3, CCR6, and CCR4 expression in CD57/PD-1 and CD151/CD99 T_FH_ populations with key to identify T_FH_ populations distinguished by CD57 and PD-1, and T_FH_ populations (Q1-Q4) distinguished by CD151 and CD99. (**h**) gMFI expression of T_FH_ markers (PD-1, ICOS, OX40), chemokine receptor markers (CXCR3, CCR6, CCR4), and novel markers (CD127, CD99, CD71, CD151, CD43, CD69) with (+) and without (-) 4 hr PMA and ionomycin stimulation. Data show experiments of 5 PBMC samples per group and 6 tonsil samples per group and mean ± SEM. Statistical tests: one-way ANOVA of multiple comparisons. **** P values are <0.0001 or otherwise indicated. Source Data

**Extended Data Fig. 10:**
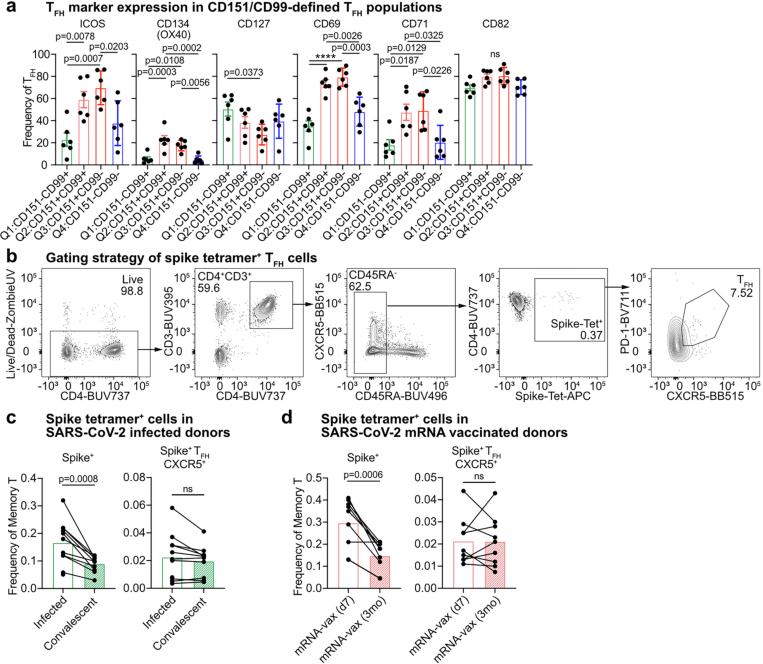
Novel markers identify distinct human T_FH_ subpopulations in spike tetramer-specific PBMCs of SARS-CoV-2 infected and mRNA vaccinated donors. **a**, CD3^+^CD4^+^CD45RA^−^CD45RO^+^CXCR5^+^ T_FH_ cells from six tonsil samples (n = 6) from healthy adult donors displaying T_FH_ surface marker frequencies in T_FH_ populations (Q1-Q4) distinguished by CD151 and CD99. **b**, Gating strategy to identify SARS-CoV-2 spike tetramer^+^ cT_FH_ cells. **c**,**d** Spike tetramer^+^ cells in (**c**) SARS-CoV-2 infection (11/11 donors; n = 11) or 6 months convalescence (10/11 donors; n = 10), or (**d**) nine COVID-19 mRNA vaccinated donors (n = 9) at 7 days post-vaccine and 3 months post-vaccine. Frequency Spike tetramer^+^ CD3^+^CD4^+^CD45RA^−^ T cells and CD3^+^CD4^+^CD45RA^−^CXCR5^+^ T_FH_ cells. Data show experiments of 8-11 samples per group. Statistical test: Two-tailed paired Student’s *t* test. **** P values are <0.0001 or otherwise indicated. Source Data

We next sought to identify a tractable method to identify cytokine-directed T_FH_ cell phenotypes within human lymphoid tissue. Using surface protein analysis in our scRNA-seq dataset, key markers of cell identity (CD4 and CD45RO) and T_FH_ (CD279 and CD278) and T_eff_ (CD62L and KLRG1) cell core signatures were identified (Extended Data Fig. 9a and Supplementary Table 2; Donor Table 2). CXCR3, CCR4 and CCR6 expression were minimally detected and did not indicate distinct T_FH_ cell clusters (Fig. 7a and Extended Data Fig. 9b)^9,55^. Instead, several markers discriminated either individual or groups of T_FH_ cell transcriptional phenotypes (Extended Data Fig. 9c). We next examined whether these markers could identify populations in human tonsils and peripheral blood mononuclear cells (PBMCs) from healthy adult donors. Consistent with previous studies, CD57^+^ T_FH_ cells were exclusively found in tonsillar samples, aligning them with a GC T_FH_ cell population with high CXCR5, ICOS and CCR4 expression to engage B cells (Fig. 7b and Extended Data Fig. 9d,e)^9,56–58^. Similarly, PD-1, CD69 and CD82 were differentially expressed between tonsil T_FH_ and cT_FH_ cells (Fig. 7c). In contrast, CD127 (IL-7Rα), CD99, CD71 and CD151 exhibited considerable overlap, albeit with different frequencies, between tonsillar PD-1^+^CD57^+^, PD-1^+^CD57^−^, PD-1^−^CD57^−^ T_FH_ cells and PD-1^−^CD57^−^ cT_FH_ cells (Fig. 7d,e and Extended Data Fig. 9f,g). T_FH_ phenotype marker expression did not align with the expression of CXCR3, CCR4 or CCR6, and their expression was largely stable following PMA/ionomycin stimulation (Extended Data Fig. 9g,h). Matching markers onto scRNA-seq clusters (C3, C7, C9: CD57; C1: CD99; C8: CD71, CD151; and C10: CD127) identified the dominant T_FH_ cell phenotype represented by each marker (Fig. 7f and Extended Data Fig. 9c). We next assessed how these markers of T_FH_ cell heterogeneity track across different tissue sites, comparing donor-matched tonsil, adenoid and PBMCs in five juvenile donors (Supplementary Table 2; Donor Table 3). While some cell surface markers (PD-1, CD57 and CD69) were restricted to lymphoid organs, CD151-, CD99-, ICOS-, CD71- and CD43-expressing T_FH_ cells had counterparts represented in each site, potentially indicating shared origins of populations (Fig. 7g). Tonsil T_FH_ cell populations defined by CD151 and CD99 exhibited distinct expression of activation and co-stimulatory molecules, suggesting distinct functional potential (Extended Data Fig. 10a). Therefore, we next explored CD151 and CD99 expression longitudinally on SARS-CoV-2 Spike tetramer^+^ antigen-specific cT_FH_ cells following either infection or mRNA-LNP (Comirnaty) vaccination (Extended Data Fig. 10b–d and Supplementary Table 2; Donor Tables 4 and 5). cT_FH_ PD-1 expression decreased following antigen clearance in convalescent infection and 3 months after vaccination (Fig. 7h,i). While no significant differences were observed using CXCR3 and CCR6, CD99^−^ (both Q3 CD151^+^ and Q4 CD151^−^) cT_FH_ cells were increased during acute SARS-CoV-2 infection compared to convalescence, which showed a shift toward Q1 (CD151^−^CD99^+^) (Fig. 7h). In comparison, Spike tetramer^+^ cT_FH_ cells 7 days after vaccination increased Q2 (CD151^+^CD99^+^), demonstrating the ability to identify distinct cT_FH_ populations following different immune challenges (Fig. 7h,i). Combined, this paired cell surface and transcriptional resource of T_FH_ phenotypes paves the way for investigating how functional T_FH_ heterogeneity arises and shapes B cell responses in human health and antibody-mediated disease.

## Discussion

Here, we interrogated the T_FH_ cell phenotypes that emerged following diverse pathogen challenge to understand how functional T_FH_ cell flexibility is established. Our data are consistent with an initial bifurcation between T_FH_ and T_eff_ cells (comprising T_H_1, T_H_2 and T_H_17 cell populations) to establish a core T_FH_ cell transcriptional signature irrespective of the pathogen class^13^. While graded T-bet expression correlated with T_eff_:T_FH_ cell ratios, the T_FH_ cell core signature was independent of *Tbx21*, *Gata3* and *Rorc*. Instead, *Foxo1*, *Bach2* and *Foxp1* opposed the T_FH_ program independently of pathogen class^39–42^. We identified several new core transcriptional regulators that may direct T_FH_ cell differentiation or function independently from the *Bcl6* transcriptional network^50–54,59^. Our data highlight a secondary differentiation branching that occurs after, or parallel with, the initial bifurcation, which directs the functional diversification of T_FH_ cells. Here, Bcl-6 was coexpressed with distinct lineage-defining transcription factors that superimpose a stimulus-specific program to direct the function and tailoring of B cell responses. Of note, some T_FH_ cell core signature genes were also found within specific T_FH_ cell phenotypes, suggesting these factors are expressed in a spectrum (rather than on/off switches). In addition to T_eff_ and T_FH_ cells, equivalent transcriptional modules led by T-bet, GATA3 and RORγt exist for T regulatory, innate lymphoid and dendritic cells^60–62^. Thus, our results highlight the dual transcriptional processes of lineage identity and functional transcriptional modules as central processes that underpin how the immune system adapts to diverse environmental challenges.

The concentrations of cytokines, namely, IL-2, IL-6 and IL-12, mediate the balance of Bcl-6, T-bet and GATA3 to direct differentiation to either T_FH_ or T_eff_ cell fates^14,17,18,21,63^. We extend this concept to show that local cytokine exposure also specifies functional T_FH_ cell phenotypes. TGFβ or IFN-I signaling altered T_FH_ cell phenotype differentiation in a context-dependent manner, and in turn, shapes B cell output. Notably, cytokine signaling was a stronger driver of T_FH_ cell differentiation than pathogen class, as T_FH_ cells in LCMV and *T.* *muris* featured IFN-I signaling pathways, whereas T_FH_ cells in influenza did not. Our mouse resource was generated using the C57BL/6 strain; however, genetic background will impact the cytokine milieu that alter T_FH_ cell specification. Nevertheless, these cytokine signaling pathways were confirmed in human T_FH_ cell populations, indicating their potential to drive the ontogeny of T_FH_ cell heterogeneity in both species. Accordingly, IFNAR deficiency mirrored T-bet deletion in mice and human T-bet loss-of-function mutations^34,64^. There are likely other cytokines that also shape T_FH_ cell function. Indeed, *H.* *polygyrus* T_FH_ cells were not enriched for either IFN-I or TGFβ signaling, suggesting that an alternative cytokine or receptor‒ligand pathway performs T_FH_ cell functions in this context. In human T_FH_ cells, we identified clusters enriched for IL-23, IL-6, IL-2 and IL-1 signaling. Thus, the local cytokine environment guides the generation of functionally distinct T_FH_ cell phenotypes. These data rationalize multiple studies that have identified conflicting or context-dependent cytokines and transcription factors that modulate T_FH_ cell differentiation, suggesting that these factors may modify the differentiation of specific T_FH_ cell phenotypes rather than the overall T_FH_ cell fate^17,22,34,65,66^.

To gain an expansive understanding of T_FH_ cell heterogeneity, we analyzed pathogen infections that differ by infection route, lymph node drainage and varied antigen load and affinity. Each of these factors likely plays a role in generating the cytokine milieu that determines the T_FH_ cell phenotype and function. Still, pathogens (*C.* *rodentium*, *T.* *muris* and *H.* *polygyrus*) infected via the same route yielded distinct T_FH_ cell phenotypes suggesting T_FH_ cell diversity is more directed by distinct pathogens and cytokine signaling than infection route alone. Even so, our dataset provides a resource for these aspects to be investigated individually. Similarly, pathogen-induced T_FH_ cell phenotypes were compared at a single time point corresponding to early GC establishment. As T_FH_ cell phenotypes may change over time^11,33^, how the stability and plasticity of T_FH_ cell signatures is maintained over the course of GC duration, memory and rechallenge responses remains to be determined. The combination of our mouse and human transcriptional dataset and human cell surface resources provides new tools to address these questions.

Although the cytokine signatures overlapped in our mouse and human T_FH_ cell datasets, there are some important differences. We showed that CD99 is a new marker for human T_FH_ cell heterogeneity, which is differentially expressed in vaccine and infection. While there is a proposed mouse homologue for CD99, this is encoded on a different chromosome (chromosome 4, rather than X and Y in humans) and was not detected in our mouse dataset. Similarly, B3GAT1 (encoding CD57) which is expressed on GC T_FH_ cells in humans^56^ did not appear in our mouse dataset. Future work will investigate the functional relevance of human T_FH_ cell phenotypes that are defined by these new markers.

We demonstrated that cytokine-mediated alterations in the T_FH_ cell phenotype influenced GC B cell and antibody production. Evidence of cytokine-biased T_FH_ cell phenotypes in human and mouse systems enables opportunities to target these signaling pathways to direct B cell and antibody responses for the development of optimal humoral immunity in response to vaccines against a diverse range of pathogens. Furthermore, our approach to match transcriptional T_FH_ cell phenotypes with novel cell surface markers of T_FH_ cell heterogeneity unlocks avenues to understand and treat antibody-mediated diseases such as immunodeficiency, allergy, asthma and autoimmunity.

## Methods

### Mice

The mice were bred and maintained on a C57BL/6 background under specific-pathogen-free conditions in-house at the Walter and Eliza Hall Institute of Medical Research (WEHI) at 19–24 °C, 45–65% humidity on a 12-h light–dark cycle. The T-bet-ZsGreen reporter^35^, IL-4–AmCyan–IL-13–DsRed–IFN-γ–GFP reporter^7,67^, IL-21–GFP reporter^10^, FoxP3–RFP reporter^68^, *Tgfbr2*-Lck^Cre^ (ref. ^69^) and *Ifnar*^−*/*−^ (ref. ^70^) mice used have been previously described. Mixed chimeras were generated via lethal irradiation of Ly5.1xC57BL/6 mice (two doses of 0.55 Cy), reconstituted with *Tgfbr2*-Lck^Cre^ or *Ifnar*^−*/*−^ and Ly5.1/2 bone marrow at a 1:1 ratio and left for 8 weeks before infection. All experiments were conducted in compliance with the guidelines of the Walter and Eliza Hall Institute Animal Ethics Committee and performed on sex-matched 6–10-week-old mice.

### Infections

Mice inoculated intravenously with 3 × 10^3^ plaque-forming units (p.f.u.) of LCMV-Armstrong were collected 12 days post-infection. Mice intranasally infected with 1 × 10^4^ p.f.u. influenza A virus strain HKx31 were collected 10 days post-infection. Mice infected via oral gavage with 2 × 10^9^ colony-forming units of *C.* *rodentium*, 200 embryonated *T.* *muris* eggs, or 200 L3 stage *H.* *polygyrus* larvae were collected 12, 21 and 12 days after each infection, respectively.

### Human tonsil, adenoid tissue and PBMC samples

Details of cryopreserved tonsil, adenoid tissue and PBMCs used in scRNA-seq and flow cytometric analyses provided in Supplementary Tables 1–5. Juvenile tonsil, adenoid tissue and PBMC sample collection were approved by the Tasmanian Human Research Ethics Committee. SARS-CoV-2 study protocols were approved by the University of Melbourne Human Research Ethics Committee (approval nos. 2056689, 13793 and 23497) and Royal Melbourne Hospital Ethics Committee (study no. 2021/272) and carried out in accordance with the approved guidelines^71^. Patients recovered from SARS-CoV-2 infection and/or been vaccinated with Moderna BA.1 bivalent messenger RNA vaccine were recruited through contacts with the investigators and invited to provide blood samples. No statistical methods were used to predetermine sample sizes. Whole blood was collected with sodium heparin anticoagulant. PBMCs were isolated via Ficoll-Paque (Sigma), cryopreserved in 90% fetal calf serum (FCS)/10% dimethyl sulfoxide (DMSO) and stored in liquid nitrogen. All procedures involving human participants were approved by and in accordance with the ethical standards of the Human Research Ethics Committee at WEHI and the 1964 Helsinki Declaration and its later amendments.

### Method details

#### Flow cytometry

All mice were collected at the early peak of T_FH_ and GC B cell accumulation in the respective draining lymph nodes for each infection. Single-cell suspensions of the appropriate draining lymph nodes were stained for surface antigen expression via the indicated antibodies for 20 min at 4 °C, followed by viability dye staining for 10 min at 4 °C. For cytokine detection, single-cell suspensions were stimulated in round-bottom tubes at 37 °C + 5% CO_2_ in RPMI with 100 ng ml^−1^ PMA (Sigma), 500 ng ml^−1^ ionomycin (Sigma), 100 ng ml^−1^ brefeldin A (BD), 100 ng ml^−1^ Monesin (BD) and 10% FCS for 4 h. Cytokine staining was performed using BD Cytofix/Cytoperm kit (BD). Transcription factor staining was performed using Invitrogen Foxp3 Transcription Factor Staining kit (Thermo Fisher). Flow cytometry analysis was performed on a BD LSRFortessa X-20, BD FACSymphony A3 Cell Analyzers (BD) and Cytek Aurora (Cytek Biosciences) and analyzed using FlowJo v.10 (FlowJo).

#### Generation of peptide MHC II tetramers

Human DPB1*04:01 SSANNCTFEYVSQPFLMDLE (SARS-CoV-2 S_167–180_) biotinylated monomers were generated by NIH Tetramer Core Facility. DRB1*15:01 NLLLQYGSFCTQLNRAL (SARS-CoV-2 S_751–767_), DRB1*04:01 YQTSNFRVQPTESIVRFPNI (SARS-CoV-2 S_313–332_), DRB1*04:01 NFSQILPDPSKPSKRSFIED (SARS-CoV-2 S_801–820_) biotinylated monomers were produced by ProImmune. Biotinylated monomers were tetramerized by sequential addition of streptavidin-APC (Thermo Fisher).

#### Tetramer staining

Cryopreserved PBMCs were thawed in RPMI-1640 (Thermo Fisher) with 10% FCS and 2% penicillin–streptomycin (RF10). Up to 1 × 10^6^ PBMCs were washed in 2% FCS/PBS before incubation with 50 nM dasatinib (in 2% FCS/PBS) at 37 °C for 30 min. Tetramers were added at 4 µg ml^−1^ at 37 °C for 60 min. A cocktail of chemokine receptors comprising of CXCR5-BB515, CXCR3-Pacific Blue and CCR6-BV785 was added during the last 30 min of tetramer staining. Cells were washed in PBS, stained with viability dye, and incubated for 30 min at 4 °C with the indicated surface stain antibodies. Cells were washed with 2% FCS/PBS and fixed with 1% Cytofix (BD). Data were acquired on a FACS Symphony A5 SE (BD).

#### Immunofluorescence staining and confocal microscopy

Lymph nodes were collected and fixed in 4% paraformaldehyde (Sigma) for 8 h at 4 °C, immersed in 30% sucrose overnight at 4 °C, and embedded in OCT compound (Tissue-Tek). The tissues were cut via a microtome (Leica) into 12–20-μm sections and mounted onto Superfrost Plus slides. The slides were incubated in blocking buffer containing 2% normal rat serum (Jackson ImmunoResearch) and 0.1% Triton X-100 (Sigma) in PBS for 24 h at 4 °C. Sections were stained with antibodies in buffer containing 0.2% normal rat serum (Jackson ImmunoResearch) and 0.01% Triton X-100 (Sigma) in PBS overnight at 4 °C. The slides were washed by immersion in PBS containing 0.1% Triton X-100 (Sigma), and the coverslips were mounted with Prolong Diamond (Thermo Fisher). Images were acquired on an LSM980 confocal microscope (Carl Zeiss MicroImaging) and processed via Zen Black (Zeiss) software.

#### Serum cytokine bead array

Blood was collected from infected mice and centrifuged at 20,000*g* for 15 min. Serum supernatant was collected and loaded onto a BD Mouse T_H_1/T_H_2/T_H_17 CBA kit (BD) according to the manufacturer’s instructions. In brief, serum and standards were added to cytokine capture beads and PE detection reagent in a 96-well plate for 2 h at 20 °C in the dark. The plate was washed, resuspended and acquired on a BD LSRFortessa X-20 cell analyzer (BD). Data analysis and standard curve generation were performed with FCAP Array Software v.3.0 (BD).

#### ELISA

High-binding 96-well ELISA plates (Sarstedt) were coated with anti-IgG unconjugated antibodies (Southern Biotech) and incubated overnight at 4 °C, followed by incubation with blocking buffer containing 1% bovine serum albumin (BSA) in PBS for 1 h at 20 °C. The supernatant was removed, and the plates were washed with PBS containing 0.04% Tween 20 (Thermo Fisher) followed by incubation with dH_2_O. Serum samples were diluted in 1% BSA in PBS, serially diluted threefold and incubated for 3 h at 37 °C. The plates were washed and incubated with secondary antibody conjugated to horseradish peroxidase (Southern Biotech) for 1.5 h at 37 °C. The plates were washed and the OPD color reaction mixture (Sigma) was added to each well. The absorbance was measured at 450 nm via a FLUOstar Omega Microplate reader (BMG LABTECH).

#### Antibodies and dyes for staining

##### Mouse T cell analysis

Single-cell suspensions were stained with fixable viability stain 700 (1:1,000 dilution; BD; cat. no. 564997); anti-CD4 (1:600 dilution; clone GK1.5; BD); anti-CD3 (1:200 dilution; clone 145-2C11; BD, cat. no. 564298); anti-CD44 (1:200 dilution; clone IM7; BD; cat. no. 560568); anti-Ly6C (1:400 dilution; clone HK1.4; BioLegend; cat. no. 128033); anti-CXCR5 (1:200 dilution; clone L138D7; BioLegend; cat. no. 145513); anti-CXCR5 (1:200 dilution; clone L138D7; BioLegend; cat. no. 145517); anti-CD162 (1:800 dilution; clone 2PH1; BD; cat. no. 740746); anti-PD-1 (1:200 dilution; clone RMP1-30; BioLegend; cat. no. 109116); anti-CD62L (1:400 dilution, clone MEL-14; Thermo Fisher; cat. no. 25-0621-82); anti-CD127 (1:200 dilution; clone SB/199; BD; cat. no. 612841); anti-IFNγ (1:400 dilution; clone XMG1.2; BD; cat. no. 557649); anti-IL-4 (1:400 dilution; clone 11B11; BD; cat. no. 554436); anti-IL-17A (1:400; clone TC11-18H10; BD; cat. no. 560220); anti-Bcl-6 (1:400 dilution; clone K112-91; BD; cat. no. 563363); anti-CXCR3 (1:200 dilution; clone CXCR3-173; BioLegend; cat. no. 126531) and anti-CCR6 (1:200 dilution; clone 29-2L17; BioLegend; cat. no. 129814).

##### Human T cell analysis

Single-cell suspensions were stained with fixable viability stain Zombie UV (1:1,000 dilution; BioLegend; cat. no. 423107); anti-CD4 (1:20 dilution; clone SK3; BD; cat. no. 612749); anti-CD4 (1:20 dilution; clone SK3; BD; cat. no. 341095); anti-CD3 (1:20; clone SK7; BD; cat. no. 564001); anti-CD8 (1:20 dilution; clone SK1; BD; cat. no. 664530); anti-CD45RA (1:50 dilution; clone HI100; BD; cat. no. 750258); anti-CD45RA (1:50 dilution; clone HI100; BD; cat. no. 560675); anti-CD45RO (1:20 dilution; clone UCHL1; BioLegend; cat. no. 304210); anti-CD45RO (1:20 dilution; clone UCHL1; BioLegend; cat. no. 304226); anti-CD27 (1:20 dilution; clone I128; BD; cat. no. 562656); anti-PD-1 (1:20 dilution; clone EH12.2H7; BioLegend; cat. no. 329928); anti-CXCR5 (1:20 dilution; clone RF8B2; BD; cat. no. 564624); anti-ICOS (1:20 dilution; clone ISA-3; Invitrogen; cat. no. 46-9948-42); anti-OX40 (1:20 dilution; clone ACT35; BioLegend; cat. no. 350025); anti-CD25 (1:20 dilution, clone BC96; BioLegend; cat. no. 302631); anti-CD127 (1:50 dilution, clone A018D5; BioLegend; cat. no. 135043); anti-CD162 (1:20 dilution, clone KPL-1; BioLegend; cat. no. 328813); anti-CXCR3 (1:20 dilution; clone G025H7; BioLegend; cat. no. 353723); anti-CCR6 (1:20 dilution; clone G034E3; BioLegend; cat. no. 353405); anti-CCR4 (1:20 dilution; clone L291H4; BioLegend; cat. no. 359439); anti-CD57 (1:20 dilution; clone QA17A04; BioLegend; cat. no. 393329); anti-CD151 (1:20 dilution; clone 50-6; BioLegend; cat. no. 350407); anti-CD71 (1:20 dilution; clone CY1G4; BioLegend; cat. no. 334119); anti-CD69 (1:20 dilution; clone FN50; BioLegend; cat. no. 310907); anti-CD82 (1:20 dilution; clone ASL-24; BioLegend; cat. no. 342109); anti-CD43 (1:20 dilution; clone CD43-10G7; BioLegend; cat. no. 343205); anti-TGFBR2 (1:20 dilution; clone FAB2411N; R&D Systems; cat. no. FAB2411N-025) and anti-CD99 (1:20 dilution; clone 3B2/TA8; BioLegend; cat. no. 371311).

##### B cell analysis

Single-cell suspensions were stained with fixable viability stain 700 (1:1,000 dilution; BD; cat. no. 564997); anti-B220 (1:800 dilution; clone RA3-6B2; BD; cat. no. 563103); anti-CD138 (1:400 dilution; clone 281-2; BD; cat. no. 563193); anti-CD95 (1:600 dilution; clone JO2; BD; cat. no. 557653); anti-IgD (1:200 dilution; clone 11-26c; WEHI Antibody Facility); anti-CD38 (1:600 dilution; clone 90; eBioscience; cat. no. 46038182); anti-CD86 (1:200 dilution; clone GL1; BD; cat. no. 563055); anti-CXCR4 (1:200 dilution; clone 2B11; Invitrogen; cat. no. 12-9991-82); anti-IgG1 (1:200 dilution; clone X56; BD; cat. no. 742480) and anti-IgG2a/2b (1:200 dilution; clone R2-40; BD; cat. no. 553399).

##### Single-cell sorting for mouse bulk RNA-seq

Single-cell suspensions were stained with fixable viability stain (1:1,000 dilution; BD, cat. no. 564406), anti-CD4 (1:600 dilution; clone GK1.5; BD; cat. no. 569845), anti-CD44 (1:200 dilution; clone IM7; BD; cat. no. 560568), anti-CXCR5 (1:200 dilution; clone L138D7; BioLegend; cat. no. 145513) and anti-PD-1 (1:200 dilution; clone RMP1-30; BioLegend; cat. no. 109121) antibodies.

##### Single-cell sorting of human tonsils

Single-cell suspensions were stained with fixable viability stain (1:1,000 dilution; BD; cat. no. 564997); anti-CD3 (1:20; clone SK7; BD; cat. no. 564001); anti-CD4 (1:20 dilution; clone SK3; BioLegend; cat. no. 344615); anti-CD8 (1:20 dilution; clone SK1; BioLegend; cat. no. 344739); anti-CD45RA (1:50 dilution; clone HI100; Invitrogen; cat. no. 25-0458-42); anti-CD45RO (1:20 dilution; clone UCHL1; BioLegend; cat. no. 304210); anti-CXCR5 (1:20 dilution; clone RF8B2; BD; cat. no. 564624); and anti-CD27 (1:20 dilution; clone I128; BD; cat. no. 562656).

##### Confocal

Lymph node tissue sections were stained with anti-CD4 (1:100 dilution; clone GK1.5–7; WEHI Antibody Facility), anti-IgD (1:200 dilution; clone 11-26c; eBioscience; cat. no. 48-5993-82) and anti-GL7 (1:100 dilution; clone GL7; BioLegend; cat. no.144606).

#### Preparation of mouse lymph node samples for bulk RNA sequencing

##### Sample preparation and cell sorting

Single-cell suspensions from draining lymph nodes of female IL-21–GFP–FoxP3–RFP reporter mice were stained for surface antigen expression via the indicated antibodies for 30 min at 4 °C, followed by viability dye staining for 15 min at 4 °C. Sample suspensions were sorted via a BD FACSAria Fusion Flow Cytometer (BD) to isolate CD4^+^CD44^+^PD-1^+^CXCR5^+^FoxP3–RFP^−^IL-21–GFP^+^ T_FH_, CD4^+^CD44^+^PD-1^+^CXCR5^+^FoxP3–RFP^−^IL-21–GFP^−^ T_FH_, CD4^+^CD44^+^PD-1^+^CXCR5^+^FoxP3–RFP^+^IL-21–GFP^+^ T_FR_, and CD4^+^CD44^+^PD-1^−^CXCR5^−^FoxP3–RFP^−^IL-21–GFP^−^ T_EFF_ cells. Biological replicates for each infection consisted of 2–3 independent experiments of 7–10 mice pooled per replicate, sorted for T_FH_, T_FR_ and T_eff_ populations.

##### Bulk RNA sequencing

RNA was isolated via a RNeasyPlusMicro kit (QIAGEN) and library was prepared via a SMART-seqPLUS Kit (Takarabio). The RNA was sequenced via the Illumina NextSeq 500 System on a HiSeq paired-end run. Sequencing reads were aligned to the GRCm39 *Mus* *musculus* reference genome release v.103, and featureCounts^72^ was subsequently used to quantify transcripts per gene. All samples were sequenced at the same time, avoiding the need for batch correction.

##### Data processing

Counts from two technical replicates (sequencing runs) were summed. Genes with low expression were determined via the edgeR::filterByExpr function (v.3.34.0)^73^ and samples with small library sizes (<1 million reads) were discarded. Relative log expression plots and PCA were used to identify variations in the data and identify batch effects. Outliers and samples with poor RNA quality were subsequently removed. Normalization was performed via the trimmed mean of log expression ratios (TMM) method^74^ with no batch effects identified.

##### Differential expression analysis

DE analysis was performed via the voom-limma pipeline^75,76^ from the limma R/Bioconductor package (v.3.48.0)^76^. Linear models were fit via limma::lmfit to a design matrix accounting for biological factors of interest against the log expression of each transcript to identify DE between contrasts. A *t*-test relative to a threshold (TREAT) criterion was then applied^77^ (with a minimal fold change threshold of 1.1) to perform statistical tests. The decideTests function was used to determine the DE genes with statistical significance *P* ≤ 0.05 and multiple testing adjustments were calculated using the Benjamini–Hochberg method with FDR at 5%.

##### Deriving the core T_FH_ cell signature

Differential expressed genes between the T_FH_ and T_eff_ cells from all the infections was derived and differential expressed genes between the T_FH_ and T_eff_ cells for each of the five individual infections was obtained. The core T_FH_ signatures were defined as the DE genes occurring in three or more infections and were present in the T_FH_ versus T_eff_ comparison from all the infections.

##### Deriving the T_FR_ cell signature

The T_FR_ signature was derived from the common differential expressed genes between the contrasts T_FR_ versus T_FH_ cells and T_FR_ versus T_eff_ cells from all infections.

##### Deriving the pathogen-specific T_FH_ cell signatures

Pathogen-specific T_FH_ signatures were derived from DE analysis results of only T_FH_ cells; specifically, the differential expressed genes between individual infections (for example LCMV) versus all other infections (influenza, *T.* *muris*, *H.* *polygyrus* and *C.* *rodentium*).

##### Deriving the pathogen-specific T_eff_ cell signatures

Pathogen-specific T_eff_ cell signatures were derived from DE analysis results of only T_eff_ cells; specifically, the differential expressed genes between individual infections (for example LCMV) versus all other infections (influenza, *T.* *muris*, *H.* *polygyrus* and *C.* *rodentium*).

##### Gene set enrichment analysis

The enrichment of marker gene sets from published T_FH_ and GC B cell datasets^36,78–84^ and precursors of exhausted and memory T cell^85–88^ datasets in the DE results was calculated using the fgsea package^89^ and visualized as NES dot plots and barcode plots for the specified gene sets^53,54,59,78,90^.

##### Visualization of signatures

DE between contrasts was visualized with MA Bland–Altman plots via the ggplot2 R package (https://ggplot2.tidyverse.org). Visualization of the intersection of DE between contrasts was performed via the UpSetR package^91^. Signatures were visualized via heatmaps via the ComplexHeatmap R package^92^ for cell surface receptors and transcriptional regulators. Cell surface receptor genes were identified on the basis of gene sets downloaded from the Mouse Genome Database^93^ for the cell surface (GO:0009986) and cell surface receptor signaling pathways (GO:0007166). Transcriptional regulator genes included those related to transcription factors annotated in the Immunological Genome Project (ImmGen) database^94^, the mouse tissue transcription factor atlas^95^, and gene sets downloaded from the Mouse Genome Database for transcription factor activity (GO:0003700), transcription activator activity (GO:0001216), transcription repressor activity (GO:0001217) and transcription factor binding (GO:0008134). Bcl-6 network transcriptional regulator genes in the T_FH_ cell core (without cell cycle genes) were visualized for T_FH_ versus T_EFF_ cell and T_FR_ versus T_EFF_ cell contrasts via the ggplot2 R package.

#### Preparation of human tonsil samples for cell sorting, scRNA-seq and CITE-seq

##### Sample preparation and cell sorting

The cryopreserved mononuclear cell suspensions from the tonsil samples were thawed and processed to form single-cell suspensions. The samples were stained for surface antigen expression via the indicated antibodies and TotalSeq HashTags (BioLegend) for 30 min at 4 °C, followed by viability dye staining for 15 min at 4 °C. Sample suspensions were sorted via a BD FACSAria Fusion Flow Cytometer (BD) to isolate CD3^+^CD4^+^CD45RA^−^CD45RO^+^CXCR5^+^CD27^+^ T_FH_ cells.

##### scRNA-seq and CITE-seq

Equal numbers of sorted cells from each tonsil sample were pooled and stained with TotalSeq-A Human Universal Cocktail (BioLegend) in accordance with the manufacturer’s protocols. Sequence tags of DNA-barcoded mAbs for CITE-seq were incorporated into existing scRNA-seq methods to enable simultaneous quantification of protein and gene expression by single cells. A total of 40,000 cells from the stained T_FH_ pool were subjected to 3′-tag capture via the 10x Genomics Chromium Controller. Library preparation was conducted for sequencing according to the manufacturer’s protocols. Next-generation sequencing was performed using the Illumina NovaSeq 6000 System. All samples were sequenced at the same time, avoiding the need for batch correction.

##### Data processing and demultiplexing of scRNA-seq data

Reads from each capture were processed via 10x Genomics Cell Ranger software (v.7.0.0). First, cellranger::mkfastq and bcl2fastq (v.2.19.1) were used to convert to FASTQ files for the gene expression (GEX), antibody-derived tag and hashtag oligonucleotide (HTO) libraries and cellranger::multi was used to generate count matrices. The GEX data were mapped to the GRCh38 human reference genome. The DropletUtils R/Bioconductor package (v.1.18.1) was used to load the Cell Ranger output files into R (v.4.2.1)^96^. For demultiplexing, the demuxmix (v.1.0.0) R/Bioconductor package^97^ was applied to the HTO data, with the ‘naive’ model and default parameters.

#### Analysis of scRNA-seq data

##### Quality control, clustering and phenotype scoring

The demultiplexed scRNA-seq data were assessed with low-quality cells, cells with high mitochondrial genes (≥20%), doublets and cells with unknown HTO tags removed. The unstimulated samples were extracted, leaving 27,022 cells and were normalized using scran^98^ and scuttle^99^ R packages and converted to log-normalized counts. The top 2,000 highly variable genes were used to perform PCA dimension reduction. The data were clustered via the Louvain method for *k* = 9 nearest neighbors and the Jaccard weighting scheme (using igraph and scran), resulting in 11 clusters. The ranked scores for the T_FH_ cell signatures were calculated using singscore^100,101^ R package. Ranked scores were visualized by overlaying across the UMAP using the scater::plotReducedDim function.

##### Differential expression analysis

DE analysis was conducted using a pseudobulked approach based on ‘clusters’ and ‘samples’ via the aggregateAcrossCells function in Scuttle^99^, after which samples with fewer than 10 cells were filtered out, and gene-level quality control was performed via edgeR::filterByExpr, leaving 33 pseudosamples and 10,595 genes for analysis. DE analyses were conducted via a voom-limma-duplicate correlation pipeline using the edgeR::voomLmFit function to fit a linear model with the cluster labels as the covariate and to estimate the consensus correlation across donors and account for donor variation as a random effect^73^. An empirical Bayes moderated *t* statistic was generated with multiple testing adjustments carried out via the Benjamini–Hochberg procedure to identify statistically significant genes (adjusted *P* < 0.05).

##### Gene set enrichment analysis

GSEA was performed using the fry approach from the limma package on gene sets from the Molecular Signature Database^102^, which includes the following categories: hallmark, curated (c2: Reactome, PID, Biocarta and KEGG) and Ontology (c5: BP and MF). Gene sets with an FDR < 0.05 were considered significant. Significant gene sets were analyzed via the vissE^103^ R package, clustering similar processes and identifying overarching biological themes. The gene set networks were generated via vissE by applying overlap coefficient thresholds of 0.25 and 0.15 (up- and downregulated, respectively) for both the C7 versus C8 and C1 versus C2 comparisons.

##### Data processing and visualization of CITE-seq data

CITE-seq data were extracted and normalized by applying the CLR (centered log ratio transformation) method across cells using from the Seurat::NormalizeData function^104^. The log count expression for selected markers was visualized by overlaying over the scRNA-seq generated UMAPs of the tonsil T_FH_ cells.

#### Significance score ranking of the human datasets

##### Data download and processing for scRNA-seq data

Publicly available scRNA-seq data from the following datasets were downloaded:

- Nehar-Belaid et al. 2020 (ref. ^105^) processed Seurat objects obtained from the authors.
- Alladina et al. 2023 (ref. ^106^), from the GSE193816 repository.
- Monian et al. 2022 (ref. ^107^), from the GSE158667 repository.
- Schattgen et al. 2024 (ref. ^108^) extracted the h5 object and metadata for 2-year T cells from https://zenodo.org/records/6476021 (ref. ^109^) to rebuild the Seurat object.
- Borcherding et al. 2024 (ref. ^110^) downloaded the Seurat object for Fig. 2 from the interactive online tool CellPilot (https://cellpilot.emed.wustl.edu) provided by the authors.
- Dooley et al. 2023 (ref. ^111^) download the Seurat object from https://zenodo.org/records/6973241 (ref. ^112^).

For preprocessing, cells were filtered via functions perCellQCMetrics and quickPerCellQC from the Scuttle R package. For the data object of Schattgen et al., scran normalization was applied (functions quickCluster, computeSumFactors and logNormCounts)^109^. The filtered cells were then pseudobulked on relevant factors including cell type. The pseudobulked samples were then filtered via edgeR::filterByExpr. Pseudobulked samples with low cell counts were removed (cell count cutoff ranges between 10 and 30 cells, specific to each dataset) and the remaining pseudobulked samples were normalized using edgeR::calcNormFactors. The pseudobulked samples were ranked via the R package singscore on the basis of the expression of genes upregulated in each of the refined pathogen-specific signatures. The T_FH_ cell-associated samples were then extracted for each dataset for visualization.

##### Data download and processing for Visium spatial tonsil data

Visium spatial tonsil data from eight donors were downloaded from the atlas of cells in human tonsils^113^ (https://zenodo.org/records/8373756; ref. ^114^). The core T_FH_ cell signature was ranked using singscore for the expression of these genes across the whole tonsil spots. The spots in the regions annotated as ‘Germinal Center’ and ‘Proliferating Follicle’ were extracted and similarly rank scored based on the expression of genes in each of the refined pathogen-specific signatures.

##### Data download and processing for Xenium spatial lymph node data

Xenium FFPE 5k lymph node data were downloaded from https://www.10xgenomics.com/datasets/preview-data-xenium-prime-gene-expression. Spatial Experiment objects were built from the transcript, metadata and cell-based data. Quality control steps were applied, including filtering out empty cells and removing cells with either total transcript counts or detected gene counts less than 10% of the quantile of the data. The T_FH_-like cells were identified by first extracting cells annotated (by 10x Genomics) as MBCs, effector CD4^+^ T cells, T cells and CD4^+^ memory T cells and then selecting cells with a positive ranked score (scored using singscore based on the expression of genes in the core up- and downregulated signatures). These cells were then rank scored using a singscore based on the expression of genes in each of the refined pathogen-specific upregulated signatures.

### Quantification and statistical analysis

#### Quantification and statistical analysis

Statistical differences between groups in datasets with one categorical variable were evaluated by unpaired *t*-tests (two groups) or one-way ANOVA (more than two groups) corrected for multiple comparisons. Statistical differences between groups in datasets with two categorical variables were evaluated by two-way ANOVA corrected for multiple comparisons. *P* < 0.05 was considered statistically significant. *P < 0.05; **P < 0.01; ***P < 0.001; ****P < 0.0001. No statistical methods were used to predetermine sample sizes but our sample sizes are similar to those reported in previous publications^115^. Data distribution was assumed to be normal but this was not formally tested. Data collection and analysis were not performed blind to the conditions of the experiments. All experimental data are presented as mean ± s.e.m. with statistical analysis performed via Prism v.9 (GraphPad Software).

### Reporting summary

Further information on research design is available in the Nature Portfolio Reporting Summary linked to this article.

## Online content

Any methods, additional references, Nature Portfolio reporting summaries, source data, extended data, supplementary information, acknowledgements, peer review information; details of author contributions and competing interests; and statements of data and code availability are available at 10.1038/s41590-025-02258-9.

## Supplementary information

Reporting Summary

Supplementary Table 1 Supplementary Tables 1–3.

## Source data

Source Data for all figures Statistical Source Data.

## Data Availability

All experimental models and reagents will be made available upon completion of a material transfer agreement. The bulk RNA-seq data (GSE302862) and scRNA-seq data and single-cell CITE-seq data (GSE302645) have been deposited to the Gene Expression Omnibus database and are publicly available. Any additional information required to reanalyze the data reported in this paper is available from the corresponding author upon request. Source data are provided with this paper.
