# Supplementary material for: Divergent cytokine and transcriptional signatures control functional T follicular helper cell heterogeneity
Source: Nat Immunol. 2025 Sep 9;26(10):1821–35. doi: 10.1038/s41590-025-02258-9 (PMC12479364; doi:10.1038/s41590-025-02258-9)
Supplement: Supplementary file 1 — Reporting Summary [file 41590_2025_2258_MOESM1_ESM.pdf]

nature portfolio

Corresponding author(s): Joanna R. Groom

Last updated by author(s): Jul 16, 2025

Reporting Summary

Nature Portfolio wishes to improve the reproducibility of the work that we publish. This form provides structure for consistency and transparency in reporting. For further information on Nature Portfolio policies, see our Editorial Policies and the Editorial Policy Checklist.

Statistics

For all statistical analyses, confirm that the following items are present in the figure legend, table legend, main text, or Methods section.

n/a	Confirmed	
☐	☒	The exact sample size (*n*) for each experimental group/condition, given as a discrete number and unit of measurement
☐	☒	A statement on whether measurements were taken from distinct samples or whether the same sample was measured repeatedly
☐	☒	The statistical test(s) used AND whether they are one- or two-sided<br>*Only common tests should be described solely by name; describe more complex techniques in the Methods section.*
☐	☒	A description of all covariates tested
☐	☒	A description of any assumptions or corrections, such as tests of normality and adjustment for multiple comparisons
☐	☒	A full description of the statistical parameters including central tendency (e.g. means) or other basic estimates (e.g. regression coefficient) AND variation (e.g. standard deviation) or associated estimates of uncertainty (e.g. confidence intervals)
☐	☒	For null hypothesis testing, the test statistic (e.g. F, t, r) with confidence intervals, effect sizes, degrees of freedom and P value noted<br>*Give P values as exact values whenever suitable.*
☐	☒	For Bayesian analysis, information on the choice of priors and Markov chain Monte Carlo settings
☐	☒	For hierarchical and complex designs, identification of the appropriate level for tests and full reporting of outcomes
☐	☒	Estimates of effect sizes (e.g. Cohen's *d*, Pearson's *r*), indicating how they were calculated

Our web collection on statistics for biologists contains articles on many of the points above.

Software and code

Policy information about availability of computer code

Data collection	Flow cytometry analysis was performed on a BD LSRFortessa X-20 cell analyser (BD Biosciences), BD FACSymphony A3 cell analyser (BD Biosciences), BD FACSymphony A5 cell analyser (BD Biosciences) and Cytek Aurora spectral cytometer (Cytek Biosciences).<br>Confocal microscopy was performed using a LSM980 confocal microscope, with a 63x/1.4 oil objective (Zeiss).<br>Bulk RNA-sequencing perfomed using the Illumina NextSeq 500 System on a HiSeq paired-end run.<br>scRNA sequencing with CITE sequencing was performed using a 10x Chromium Next GEM Single Cell 3' (10x Genomics) and sequenced using the NovaSeq 6000 System (Illumina).
Data analysis	Flow cytometry data analysis was performed using FlowJo v10 (FlowJo, LLC) and SpectroFlo 3.3 (Cytek Biosciences).<br>Images from confocal microscopy were processing using Zen Black (Zeiss) software.<br>Graphs and statistical significance were generated using Prism 10 (GraphPad)<br>bulk RNA-seq data was processed using R package R-subread where sequencing reads were aligned to the GRCm39 Mus musculus reference genome release version 103 and transcripts per gene quantified using function featureCounts. Quality control, processing and downstream analyses conducted using R packages fgsea v1.30.0, edgeR v3.42.0, limma v3.48.0, ggplot2 v3.5.1, ComplexHeatmap v2.20.0 and UpSetR v1.4.0.<br>scRNA-seq data was processed using Cell Ranger v7 (10x Genomics), DropletUtils v1.18.1 and R v4.2.1 (R Core Team). Reads were mapped to the GRCh38 human reference genome. Downstream analyses conducted using R packages SingleCellExperiment v1.26.0, Seurat v5.1.0, scater v1.32.0, scran v1.32.0, scuttle v1.14.0, edgeR v4.2.2 and limma v3.60.6.

For manuscripts utilizing custom algorithms or software that are central to the research but not yet described in published literature, software must be made available to editors and reviewers. We strongly encourage code deposition in a community repository (e.g. GitHub). See the Nature Portfolio guidelines for submitting code & software for further information.

Data

Policy information about availability of data

All manuscripts must include a data availability statement. This statement should provide the following information, where applicable:

- Accession codes, unique identifiers, or web links for publicly available datasets
- A description of any restrictions on data availability
- For clinical datasets or third party data, please ensure that the statement adheres to our policy

The bulk RNA-seq data (GSE302862) and single-cell RNA-seq data and single-cell CITE-seq data (GSE302645) have been deposited to the Gene Expression Omnibus database and are publicly available as of the date of publication.

Research involving human participants, their data, or biological material

Policy information about studies with human participants or human data. See also policy information about sex, gender (identity/presentation), and sexual orientation and race, ethnicity and racism.

Reporting on sex and gender	Human tonsil, adenoid tissue and PBMC samples were obtained from both male and female donors.
Reporting on race, ethnicity, or other socially relevant groupings	*Please specify the socially constructed or socially relevant categorization variable(s) used in your manuscript and explain why they were used. Please note that such variables should not be used as proxies for other socially constructed/relevant variables (for example, race or ethnicity should not be used as a proxy for socioeconomic status).*<br>*Provide clear definitions of the relevant terms used, how they were provided (by the participants/respondents, the researchers, or third parties), and the method(s) used to classify people into the different categories (e.g. self-report, census or administrative data, social media data, etc.)*<br>*Please provide details about how you controlled for confounding variables in your analyses.*
Population characteristics	Cryopreserved tonsil samples for scRNAseq were obtained from 3 healthy adults (30-38 years old; 1 female, 2 male).<br>Cryopreserved PBMCs from 5 healthy adults (27-47 years old; 3 female, 2 male) and tonsil samples from 6 healthy adults (30-38 years old; 2 female, 2 male, 2 NA were obtained for flow cytometric analyses.<br>Cryopreserved tonsil, adenoid tissue and PBMCs from 5 healthy juvenile donors (4-10 years old; 2 female, 3 male) were obtained for flow cytometric analyses.<br>Cryopreserved PBMCs from 11 SARS-CoV-2 infected adults (49-76 years old; 4 female, 7 male) and PBMCs from 9 SARS-CoV-2 mRNA vaccinated adults (26-60 years old; 4 female, 5 male) were obtained for flow cytometirc analyses.
Recruitment	5 healthy adults were recruited from the Volunteer Blood Donor Registry (VBDR; WEHI). Cryopreserved tonsil samples were provided by Prof Cindy Ma (Garvan Institute). Patients who had recovered from SARS-CoV-2 infection and/or been vaccinated with Moderna BA.1 bivalent mRNA vaccine were recruited through contacts with the investigators and invited to provide a blood sample. Sample collection was based on patient availability without additional criteria applied. No self-selection bias is present. No statistical methods were used to predetermine sample sizes.
Ethics oversight	Juvenile tonsil, adenoid tissue and PBMC sample collection were approved by the Tasmanian Human Research Ethics Committee. SARS-CoV-2 study protocols were approved by the University of Melbourne Human Research Ethics Committee (approval nos. 2056689, 13793, 23497) and Royal Melbourne Hospital Ethics Committee (study number 2021/272). All procedures involving human participants were approved by and in accordance with the ethical standards of Human Research Ethics Committee at WEHI and the 1964 Helsinki Declaration and its later amendments.

Note that full information on the approval of the study protocol must also be provided in the manuscript.

Field-specific reporting

Please select the one below that is the best fit for your research. If you are not sure, read the appropriate sections before making your selection.

☒ Life sciences ☐ Behavioural & social sciences ☐ Ecological, evolutionary & environmental sciences

For a reference copy of the document with all sections, see nature.com/documents/nr-reporting-summary-flat.pdf

Life sciences study design

All studies must disclose on these points even when the disclosure is negative.

Sample size	No formal sample size power calculations were performed. We chose the standards in the field when selecting sample size. For mouse phenotyping, an n of 3 or greater and at least 10 mice total per group were used. RNA sequencing was performed on 2-3 biological replicates from individual cohorts per condition.
Data exclusions	No data was excluded from the analysis.
Replication	Mouse experiments were successfully replicated in two or more individual cohorts.

Randomization	Samples were grouped by genotype and mice in groups were infected with the same infectious isolate (LCMV Armstrong, Influenza A, Trichuris muris, Heligmosomoides polygyrus, and Citrobacter rodentium). No other randomization was performed.
Blinding	Blinding of samples of animals was not specifically used as physical attributes (such as disease scores or tumor growth) were not assessed. There was no risk of bias in this study from knowing the sample details so blinding was not relevant. Quantification of flow cytometry, sequencing and imaging data was performed in a uniform manner for all samples analyzed.

Reporting for specific materials, systems and methods

We require information from authors about some types of materials, experimental systems and methods used in many studies. Here, indicate whether each material, system or method listed is relevant to your study. If you are not sure if a list item applies to your research, read the appropriate section before selecting a response.

Materials & experimental systems

n/a	Involved in the study	
☐	☒	Antibodies
☒	☐	Eukaryotic cell lines
☒	☐	Palaeontology and archaeology
☐	☒	Animals and other organisms
☒	☐	Clinical data
☒	☐	Dual use research of concern
☒	☐	Plants

Methods

n/a	Involved in the study	
☒	☐	ChIP-seq
☐	☒	Flow cytometry
☒	☐	MRI-based neuroimaging

Antibodies

Antibodies used

Mouse T cell analysis
Single cell suspensions were stained with fixable viability stain 700 (1:1,000; BD; Cat#564997); anti-CD4 (1:600; clone GK1.5; BD); anti-CD3 (1:200; clone 145-2C11; BD, Cat#564298); anti-CD44 (1:200; clone IM7; BD; Cat#560568); anti-Ly6C (1:400; clone HK1.4; BioLegend; Cat#128033); anti-CXCR5 (1:200; clone L138D7; BioLegend; Cat#145513); anti-CXCR5 (1:200; clone L138D7; BioLegend; Cat#145517); anti-CD162 (1:800; clone 2PH1; BD; Cat#740746); anti-PD-1 (1:200; clone RMP1-30; BioLegend; Cat#109116); anti-CD62L (1:400, clone MEL-14; ThermoFisher; Cat#25-0621-82); anti-CD127 (1:200; clone SB/199; BD; Cat#612841); anti-IFN[illegible] (1:400; clone XMG1.2; BD; Cat#557649); anti-IL-4 (1:400; clone 11B11; BD; Cat#554436); anti-IL-17A (1:400; clone TC11-18H10; BD; Cat#560220); anti-Bcl-6 (1:400; clone K112-91; BD; Cat#563363); anti-CXCR3 (1:200; clone CXCR3-173; BioLegend; Cat#126531) and anti-CCR6 (1:200; clone 29-2L17; BioLegend; Cat#129814).

Human T cell analysis
Single cell suspensions were stained with fixable viability stain Zombie UV (1:1,000; BioLegend; Cat#423107); anti-CD4 (1:20; clone SK3; BD; Cat#612749); anti-CD4 (1:20; clone SK3; BD; Cat#341095); anti-CD3 (1:20; clone SK7; BD; Cat#564001); anti-CD8 (1:20; clone SK1; BD; Cat#664530); anti-CD45RA (1:50; clone HI100; BD; Cat#750258); anti-CD45RA (1:50; clone HI100; BD; Cat#560675); anti-CD45RO (1:20; clone UCHL1; BioLegend; Cat#304210); anti-CD45RO (1:20; clone UCHL1; BioLegend; Cat#304226); anti-CD27 (1:20; clone I128; BD; Cat#562656); anti-PD-1 (1:20; clone EH12.2H7; BioLegend; Cat#329928); anti-CXCR5 (1:20; clone RF8B2; BD; Cat#564624); anti-ICOS (1:20; clone ISA-3; Invitrogen; Cat#46-9948-42); anti-OX40 (1:20; clone ACT35; BioLegend; Cat#350025); anti-CD25 (1:20, clone BC96; BioLegend; Cat#302631); anti-CD127 (1:50, clone A018D5; BioLegend; Cat#135043); anti-CD162 (1:20, clone KPL-1; BioLegend; Cat#328813); anti-CXCR3 (1:20; clone G025H7; BioLegend; Cat#353723); anti-CCR6 (1:20; clone G034E3; BioLegend; Cat#353405); anti-CCR4 (1:20; clone L291H4; BioLegend; Cat#359439); anti-CD57 (1:20; clone QA17A04; BioLegend; Cat#393329); anti-CD151 (1:20; clone 50-6; BioLegend; Cat#350407); anti-CD71 (1:20; clone CY1G4; BioLegend; Cat#334119); anti-CD69 (1:20; clone FN50; BioLegend; Cat#310907); anti-CD82 (1:20; clone ASL-24; BioLegend; Cat#342109); anti-CD43 (1:20; clone CD43-10G7; BioLegend; Cat#343205); anti-TGFBR2 (1:20; clone FAB2411N; R&D Systems; Cat#FAB2411N-025) and anti-CD99 (1:20; clone 3B2/TA8; BioLegend; Cat#371311).

B cell analysis
Single cell suspensions were stained with fixable viability stain 700 (1:1,000; BD; Cat#564997); anti-B220 (1:800; clone RA3-6B2; BD; Cat#563103); anti-CD138 (1:400; clone 281-2; BD; Cat#563193); anti-CD95 (1:600; clone JO2; BD; Cat#557653); anti-IgD (1:200; clone 11-26c; WEHI Antibody Facility); anti-CD38 (1:600; clone 90; eBioscience; Cat#46038182); anti-CD86 (1:200; clone GL1; BD; Cat#563055); anti-CXCR4 (1:200; clone 2B11; Invitrogen; Cat#12-9991-82); anti-IgG1 (1:200; clone X56; BD; Cat#742480) and anti-IgG2a/2b (1:200; clone R2-40; BD; Cat#553399).

Single cell sorting for mouse bulk RNA-seq
Single cell suspensions were stained with fixable viability stain (1:1,000; BD, Cat#564406), anti-CD4 (1:600; clone GK1.5; BD; Cat#569845), anti-CD44 (1:200; clone IM7; BD; Cat#560568), anti-CXCR5 (1:200; clone L138D7; BioLegend; Cat#145513) and anti-PD-1 (1:200; clone RMP1-30; BioLegend; Cat#109121) antibodies.

Single cell sorting of human tonsils
Single cell suspensions were stained with fixable viability stain (1:1,000; BD; Cat#564997); anti-CD3 (1:20; clone SK7; BD; Cat#564001); anti-CD4 (1:20; clone SK3; BioLegend; Cat#344615); anti-CD8 (1:20; clone SK1; BioLegend; Cat#344739); anti-CD45RA (1:50; clone HI100; Invitrogen; Cat#25-0458-42); anti-CD45RO (1:20; clone UCHL1; BioLegend; Cat#304210); anti-CXCR5 (1:20; clone RF8B2; BD; Cat#564624); and anti-CD27 (1:20; clone I128; BD; Cat#562656).

Confocal
Lymph node tissue sections were stained with anti-CD4 (1:100; clone GK1.5-7; WEHI Antibody Facility), anti-IgD (1:200; clone 11-26c; eBioscience; Cat#48-5993-82) and anti-GL7 (1:100; clone GL7; Biolegend; Cat#144606) antibodies.

Validation

All antibodies used came from commercial vendors as specified above. Specificity was based on manufacturers provided description and data sheets, and previously published clones and flurochromes. Validation was provided by example staining on manufacturers website and additional validation came from references provided by each manufacturer. Antibodies were titrated on relevant cells and tissues prior to usage.

Animals and other research organisms

Policy information about studies involving animals; ARRIVE guidelines recommended for reporting animal research, and Sex and Gender in Research

Laboratory animals

T-bet-ZsGreen reporter, IL-4-AmCyan-IL-13-DsRed-IFN-γ-GFP reporter, IL-21 GFP reporter, FoxP3-RFP reporter, Tgfbr2-Lck, and Ifnar -/- mice have been previously described. Mice were bred and maintained on a C57BL/6 background under specific pathogen-free conditions in house at WEHI at 19-24°C, 45-65% humidity on a 12 h light/dark cycle. Experiments were performed on sex-matched mice of 6-10 weeks of age.

Wild animals

The study did not involve wild animals.

Reporting on sex

Both males and females were used. Individual experiments were sex matched.

Field-collected samples

The study did not involve field-collected samples

Ethics oversight

All experiments were conducted in compliance with the Walter and Eliza Hall Institute Animal Ethics Committee. All procedures involving human participants were approved by and in accordance with the ethical standards of Human Research Ethics Committee at WEHI and the 1964 Helsinki Declaration and its later amendments.

Note that full information on the approval of the study protocol must also be provided in the manuscript.

Plants

Seed stocks

Report on the source of all seed stocks or other plant material used. If applicable, state the seed stock centre and catalogue number. If plant specimens were collected from the field, describe the collection location, date and sampling procedures.

Novel plant genotypes

Describe the methods by which all novel plant genotypes were produced. This includes those generated by transgenic approaches, gene editing, chemical/radiation-based mutagenesis and hybridization. For transgenic lines, describe the transformation method, the number of independent lines analyzed and the generation upon which experiments were performed. For gene-edited lines, describe the editor used, the endogenous sequence targeted for editing, the targeting guide RNA sequence (if applicable) and how the editor was applied.

Authentication

Describe any authentication procedures for each seed stock used or novel genotype generated. Describe any experiments used to assess the effect of a mutation and, where applicable, how potential secondary effects (e.g. second site T-DNA insertions, mosiacism, off-target gene editing) were examined.

Flow Cytometry

Plots

Confirm that:

☒ The axis labels state the marker and fluorochrome used (e.g. CD4-FITC).

☒ The axis scales are clearly visible. Include numbers along axes only for bottom left plot of group (a 'group' is an analysis of identical markers).

☒ All plots are contour plots with outliers or pseudocolor plots.

☒ A numerical value for number of cells or percentage (with statistics) is provided.

Methodology

Sample preparation

Single-cell suspensions were stained for surface antigen expression using indicated antibodies for 20 minutes at 4°C, followed by viability dye staining for 10 minutes at 4°C. For cytokine detection, single-cell suspensions were stimulated in round-bottom tubes at 37°C + 5% CO_2 in RPMI with 100 ng/ml of PMA (Sigma), 500 ng/ml Ionomycin (Sigma), 100 ng/mL Brefeldin A (BD Biosciences), 100 ng/mL monesin (BD Biosciences) and 10% FCS for 4 hours. Cytokine staining was performed using the BD Cytofix/Cytoperm Kit (BD Biosciences). Transcription factor staining was performed using the Invitrogen Foxp3 Transcription Factor Staining Kit (Thermo Fisher Scientific).

Instrument

Flow cytometry analysis was performed on a BD LSRFortessa X-20 cell analyser (BD Biosciences), BD FACSymphony A3 cell analyser (BD Biosciences), BD FACSymphony A5 cell analyser (BD Biosciences), and Cytek Aurora spectral cytometer (Cytek

Biosciences).

Software | Data analysis was performed with FlowJo v10 (FlowJo LLC)

Cell population abundance | Cell sorting was performed using a BD FACSAria Fusion Flow Cytometer (BD Biosciences)

Gating strategy | All samples were pre-gated using the following strategy: 1) Lymphocytes based on FSC-A/SSC-A; 2) Singlets based on SSC-H/SSC-A; 3) Live cells based on viability dye. Clear boundaries between negative and positive samples were used to determine gating strategies. Representative plots are shown for all quantified data.

☒ Tick this box to confirm that a figure exemplifying the gating strategy is provided in the Supplementary Information.
